# Three-Dimensional Unified Motion Control of a Robotic Standing Wheelchair for Rehabilitation Purposes

**DOI:** 10.3390/s21093057

**Published:** 2021-04-27

**Authors:** Jessica S. Ortiz, Guillermo Palacios-Navarro, Víctor H. Andaluz, Luis F. Recalde

**Affiliations:** 1 Department of Electronic Engineering and Communications, University of Zaragoza, 44003 Teruel, Spain; 2 Departamento de Eléctrica y Electrónica, Universidad de las Fuerzas Armadas–ESPE, Sangolquí 171103, Ecuador; vhandaluz1@espe.edu.ec (V.H.A.); lfrecalde1@espe.edu.ec (L.F.R.)

**Keywords:** autonomous task, dynamic control, rehabilitation, robotic systems, standing wheelchair, unified motion

## Abstract

Technological advances in recent years have shown interest in the development of robots in the medical field. The integration of robotic systems in areas of assistance and rehabilitation improves the user’s quality of life. In this context, this article presents a proposal for the unified control of a robotic standing wheelchair. Considering primary and secondary tasks as control objectives, the system performs tasks autonomously and the change of position and orientation can be performed at any time. The development of the control scheme was divided in two parts: (i) kinematic controller to solve the desired motion problem; and (ii) dynamic compensation of the standing wheelchair–human system. The design of the two controllers considers the theory of linear algebra, proposing a low computational cost and an asymptotically stable algorithm, without disturbances. The stability and robustness analysis of the system is performed by analyzing the evolution of the control errors in each sampling period. Finally, real experiments of the performance of the developed controller are performed using a built and instrumented standing wheelchair.

## 1. Introduction

Within technological advances in the last decade, robotics has achieved exponential development, since robots are no longer limited to the industrial sector by performing repetitive movements in structured work environments. New robots are considered sophisticated and intelligent as they are equipped with the ability to perform tasks and interact with a high degree of autonomy in unstructured or partially structured environments [1]. This leads to the integration between robots and people through service robots, the same that aim to facilitate the user’s life by performing tasks that provide entertainment, comfort, safety, or protection. Within this group, we find robot pets that stimulate the development of children, mobile manipulators that transport heavy loads, and assistance robots that mobilize or perform rehabilitation procedures on people with health problems, among others [2,3]. According to their applications, robots can be subdivided into six main groups: *(i) Military robots*, which are used to perform tasks of exploration, surveillance, security, rescue, etc., and are responsible for carrying out activities in hostile environments that can be considered high-risk for people [4,5]; *(ii) Construction robots* have the ability to be programmed to perform complex tasks or operate semi-autonomously, aimed at improving productivity as well as accident prevention in the workplace [6,7]; *(iii) Field robots* have transformed various aspects of the agricultural, livestock, and mining industries, reducing production costs and increasing profits, thus increasing the economy of population [8,9]; *(iv) Learning robots,* which are didactic tools that improve the learning process, and they are regularly implemented in stimulation therapies for children with learning problems such as autism and attention deficit, among others [2,10,11]; *(v) Entertainment robots*, capable of performing various tasks with the aim of entertaining and accompanying human beings, they are commonly used as interactive toys for children [12]; and finally *(vi) Robots in medicine,* which can be autonomous or tele-operated. They are classified into surgeon robots, prosthetic robots, therapeutic robots, and assistance robots, considered as precise mechanisms with high accuracy due to the large number of sensors and actuators that have this technology [13,14].

Medicine has undergone tireless and accelerated evolution due to the implementation of robotics in the field of health, allowing a gigantic and favorable revolution in the attention and care of people with health problems. Thus, robotics focused on medicine has developed biomechanisms capable of interacting with patients with physical disabilities, caused by congenital, hereditary, or chromosomal factors, degenerative diseases, infectious or metabolic diseases, accidents, etc. [13,14]. One of the most common injuries that affect people is spinal cord injury (SCI), that is, an injury of different structures (osteoligamentous, cartilage, vascular muscle, meningeal, root, and medullary) of the spine at different levels. The main cause of SCI is traffic accidents (50%). There are also occupational and sports accidents [15]. Between 25% and 60% of the cases are accompanied by multiple injuries, such as head, thoracic, and pelvic, among other injuries that may be related to firearm injuries, falls from heights higher than four meters, explosive wave injuries, and diving into shallow waters [16]. Multiple methods are used for therapy of people with injuries, providing a better lifestyle for daily tasks. Thus, depending on the type of disability that the patient presents, there are robotic mechanisms that facilitate safe movements, and even most mechanisms offer a certain degree of autonomy to the person by providing motor assistance, e.g., crutches, walkers, canes, exoskeletons, and mobile chairs, among other prototypes that allow people to move around [17,18].

Nowadays, the most common robotic mechanisms for assistance and/or rehabilitation are autonomous and semi-autonomous wheelchairs or intelligent walkers [19,20,21]. According to the two-wheeled chair literature, the scientific community is working on four main aspects: *(a) Construction*. There are different proposals (commercial and non-commercial) for standing wheelchairs, which allow the user to have manual or autonomous control [22]. To perform that, the different prototypes are instrumented with intrinsic and extrinsic sensors, in order to perform fully or partially autonomous tasks for the benefit of the user. Among the different construction criteria, most authors focus mainly on the analysis of the robotic system considering the center of mass, with the aim that the standing wheelchair has a mechanical stability to perform standing tasks in a safe way for the user [23]; *(b) Modeling* is one of the topics with the greatest number of contributions, since obtaining a kinematic and dynamic model is essential for the implementation of control algorithms [24]. Thus, there are works that focus on determining a dynamic model that represents the behavior of the standing wheelchair human system. From this point of view, there are dynamic models that consider the movement of the mobile platform and the standing station as a single system. In addition, another trend is to independently analyze the movement of the mobile platform and the standing station [25]; *(c) Stabilization control*. The physical stabilization proposals mainly consider the incorporation of active mechanisms, where the seat motion is the basis of the stability control analysis of the robotic system [25,26,27]; *(d) Motion control.* The motion control schemes of a wheelchair are classified as follows:

- *Decentralized control law.* It implies controllers that work independently, i.e., a controller in charge of the moving platform and another one devoted to the standing action. From the relative angular velocity between the driving wheels of a standing wheelchair, the movement patterns of the robotic system are defined: turning, going in a straight line, or stopping. This is called a differential traction mechanism [28,29,30,31]. For standing control, movement patterns are going up and down in a straight line or staying still. Rahman et al. [31] implemented an adaptive proportional-integral-derivative(PID) control of a decentralized standing chair that considered the speed change of the robotic system.
- *Unique control law.* It is based on the use of a controller for the entire standing wheelchair robotic system. In this case, the control algorithm design considers the mobile platform of the foot station as a single robotic system [25]. Therefore, the desired task of movement of the robotic system must be considered as a single control point, which differs from the previous case, where two independent control points are considered for the mobile platform and for the standing station.

The main objective of the autonomous control of a standing wheelchair deals with the improvement of the quality of life of the users, through greater independence in movements when executing a task [32]. The independence of the robotic system is obtained through the automatic generation of tracking control, which allows following the desired trajectory of the wheelchair. The motion control of the robotic system may have three types of problems: *(i) point stabilization control*: stabilizes the wheelchair with standing considering one target point [17]; *(ii) trajectory tracking control*: whose purpose is to allow the standing wheelchair to follow a parameterized reference in time [33]; and *(iii) path-following control:* allows the robotic system to converge with desired trajectory [21].

In this work, a unified control algorithm is implemented, enabling autonomous navigation of the standing wheelchair robotic system, in order to allow a person with motor disabilities to mobilize through a trajectory of semi-structured work environments. Firstly, to implement the proposed control scheme, the behavior of the non-holonomic standing wheelchair is determined, by obtaining the kinematic and dynamic models of the chair–human system. The mathematical models obtained have a suitable structure in order to implement advanced control algorithms. The kinematic and dynamic models consider linear and angular velocities as maneuverability inputs, which is similar to real robots. On the other hand, the proposed controller consists of two subsystems: *(i) unified kinematic controller*, which solves the motion problem of the desired task, and is a controller with saturation of velocity commands; and *(ii) dynamic compensation controller,* based on the dynamic model obtained from the standing wheelchair robot, with the velocity inputs being calculated by means of the kinematic controller of the robotic system. The control algorithms proposed in this work are developed using numerical methods and linear algebra theories that allow to compute the control actions, so that the robotic standing wheelchair reaches a desired position $\left(X,Y,Z\right)$ and a desired orientation with respect to the inertial frame <R> at each sampling time $\left(t=kT_{0}\right)$. Additionally, both the stability analysis and the robustness analysis are analytically demonstrated to evaluate the stability of the proposed control scheme. Finally, to evaluate the proposed control scheme, experimental tests are carried out, which allow validating the mathematical models obtained, as well as the proposed kinematic and dynamic controllers. Finally, to evaluate the proposed control scheme, validation of the mathematical models, and kinematic and dynamic controllers obtained through experimental tests are performed.

The main contribution of this paper deals with the proposal of a cascade control scheme that considers the kinematics and dynamics of a wheelchair with bipedestation. The control strategy is based on the axioms and properties of linear algebra, as well as on the conceptualization of Markov properties, in order to determine the evolution of the control states as a function of the current states of the robotic system. The proposed unified kinematic control of the standing chair considers as inputs the desired positions and velocities of the task with respect to an inertial reference system <R>. Our unified control, unlike other works available in the literature, has the advantage of solving the standing wheelchair motion problem through the implementation of the concept of the null space of redundant systems. Furthermore, a dynamic compensation control based on the dynamic model of the human–wheelchair system is proposed. To perform that, a new dynamic model that considers as input signals the maneuvering speeds of the standing wheelchair is proposed, differing from other works found in the literature. Finally, the stability and robustness of the proposed control scheme is analyzed, ensuring that the control errors are bounded as a function of the velocity error.

## 2. Robotic Standing Wheelchair Modeling

**Figure 1:**
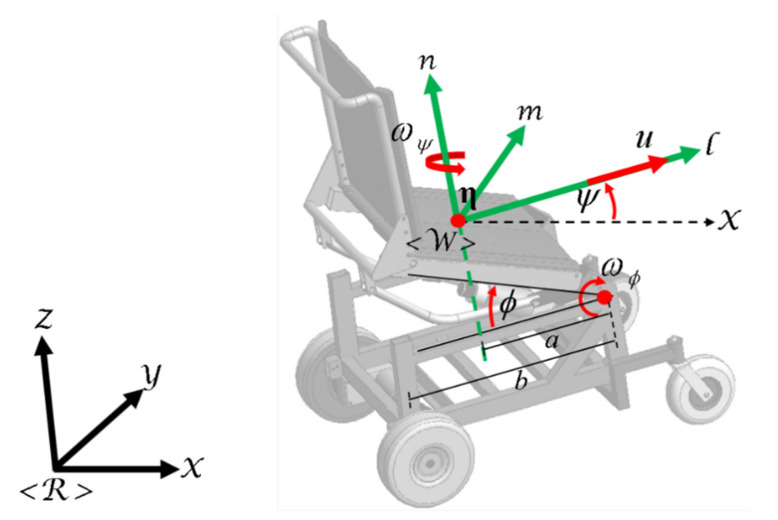
Robotic standing wheelchair with displaced point of interest $\eta \left(\eta_{x},\eta_{y},\eta_{z}\right)$.

This section presents the kinematic and dynamic model of a standing wheelchair robot. The standing wheelchair robot has a differential drive, i.e., it is a mobile robot with the ability to move and rotate independently on the vertical axis. The $\left(X,Y,Z\right)$ axis of the <R> reference frame is where the standing wheelchair robotic system moves. Traditionally, the motion control design of a mobile robot with differential drive has considered, as a point of interest, the center of the virtual axis (located between the fixed wheels of the robot) [34]. In this work, we consider a point of interest $\eta \left(\eta_{x},\eta_{y},\eta_{z}\right)$ displaced from the center of the virtual axis of the fixed wheels, so that the dynamic compensation of the robotic system delivers a real behavior, i.e., the standing wheelchair robot corrects errors generated by different external factors, such as the user’s change in posture or the friction of the different surfaces, among others [17,21]. Figure 1 illustrates the point of interest $\eta \left(\eta_{x},\eta_{y},\eta_{z}\right)$ displaced a distance “a” from the standing wheelchair robot.

### 2.1. Standing Wheelchair Kinematic Model

The position and orientation of the standing wheelchair robot is given by a vector $q\in R^{m}$ with m=4 coordinates of the <R> reference system; defined as *operating coordinates* of the standing wheelchair, where $q=[\begin{array}{cccc} \eta_{x} & \eta_{y} & \eta_{z} & \psi \end{array}]$, with $\eta_{x}$, $\eta_{y}$ and $\eta_{z}$ represent the position of the robot on the <R> reference axis; and ψ represents the orientation of the standing wheelchair. The operating space of platform M is made up of all the robot locations.

Obtaining the kinematic model of the standing wheelchair robot requires the location of the point of interest with respect to the configuration functions of the robotic system, that is, the operating coordinates of the robot must be a function of the generalized coordinates of the robot.

(1) $$\begin{array}{ll} f: & N\;\to \;M\\ & q\mapsto \;\eta =f\left(q\right) \end{array}$$

The configuration space of the robotic system is defined as N. The instantaneous kinematic model of a wheelchair standing robot provides the location of the robot with respect to the derivative of the point of interest $\dot{\eta }=\frac{\partial f\left(q\right)}{\partial q}\mu$, where the point velocity vector of interest is defined by $\dot{\eta }=[\begin{array}{cc} \begin{array}{ccc} {\dot{\eta }}_{x} & {\dot{\eta }}_{y} & {\dot{\eta }}_{z} \end{array} & \dot{\psi } \end{array}]$, and the vector μ represents the mobility control of the wheelchair robot. For this case, the kinematic model of the robot considers three velocities located in the <W> reference frame. The linear velocity u and two angular velocities $\omega_{\psi }$ and $\omega_{\phi }$ are used to guide the displacement of the wheelchair robot in the <W> reference axis. In summary, the motion of the standing wheelchair robot in the <R> reference system is defined as:

(2) $$\left\{\begin{array}{l} {\dot{\eta }}_{x}=u\cos\psi -\omega_{\psi }\left(a\sin\psi +b\left(1-\cos\phi \right)\sin\psi \right)+\omega_{\phi }b\sin\phi \cos\psi \\ {\dot{\eta }}_{y}=u\sin\psi +\omega_{\psi }\left(a\cos\psi +b\left(1-\cos\phi \right)\cos\psi \right)+\omega_{\phi }b\sin\phi \sin\psi \\ {\dot{\eta }}_{z}=\omega_{\phi }b\cos\phi \\ \dot{\psi }=\omega_{\psi } \end{array}\right.$$

where ${\dot{\eta }}_{x}$,${\dot{\eta }}_{y}$, and ${\dot{\eta }}_{z}$ are the robotic standing wheelchair-point interest velocities (whose position is being controlled) with respect to <R>; where a and b are distances measured in meters (see Figure 1). Moreover, the Equation system (2) can be written in another way as:

(3) $$\left[\begin{array}{c} {\dot{\eta }}_{x}\\ {\dot{\eta }}_{y}\\ {\dot{\eta }}_{z}\\ \dot{\psi } \end{array}\right]=\left[\begin{array}{ccc} \cos\psi & -a\sin\psi -b\left(1-\cos\phi \right)\sin\psi & b\sin\phi \cos\psi \\ \sin\psi & a\cos\psi +b\left(1-\cos\phi \right)\cos\psi & b\sin\phi \sin\psi \\ 0 & 0 & b\cos\phi \\ 0 & 1 & 0 \end{array}\right]\left[\begin{array}{c} u\\ \omega_{\psi }\\ \omega_{\phi } \end{array}\right]$$

Equation (3) can be compactly described as:

(4) $$\dot{\eta }\left(t\right)=J\left(\psi ,\phi \right)\mu \left(t\right)$$

where the vector of axis velocities of the <R> system is represented by $\dot{\eta }\left(t\right)\in R^{m}$ with m=4; and around the axis Z the angular velocity. The Jacobian matrix defined by $J\left(\psi ,\phi \right)\in R^{m\;x\;n}$ determines the linear mapping of the velocity vector $\dot{\eta }\left(t\right)$, the velocities vector of the robotic wheelchair $\mu \left(t\right)$, and the maneuverability vector of the robotic system is $\mu \left(t\right)$, defined as $\mu \left(t\right)\in R^{n}$ with n=3. For all the above, $J\left(\psi ,\phi \right)\in R^{4\;x\;3}$ implies that Equation (4) represents the behavior of a sub-powered robotic system, i.e., the number of dimensions of the standing wheelchair robot workspace is greater than the degrees of freedom of the robotic system.

The mobile platform satisfies the conditions of pure rolling and non-slipping given the non-holonomic constraint:

(5) $${\dot{\eta }}_{y}\cos\psi -{\dot{\eta }}_{x}\sin\psi -\omega_{\psi }\left(a+b\left(1-\cos\phi \right)\right)=0$$

### 2.2. Standing Wheelchair Dynamic Model

In this subsection, the dynamic modeling of the standing wheelchair robot is presented, for which a separate analysis is considered: i) Mobile platform dynamics, in which only the displacement of the standing wheelchair on the X−Y plane of system <R> is considered; and ii) Bipedestation dynamic, in which the movement of the standing wheelchair on the Z axis of the reference system <R> is considered. It is important to mention that in the process of dynamic modeling; the mass of the human is considered.

#### 2.2.1. Mobile Platform Dynamic Model

**Figure 2:**
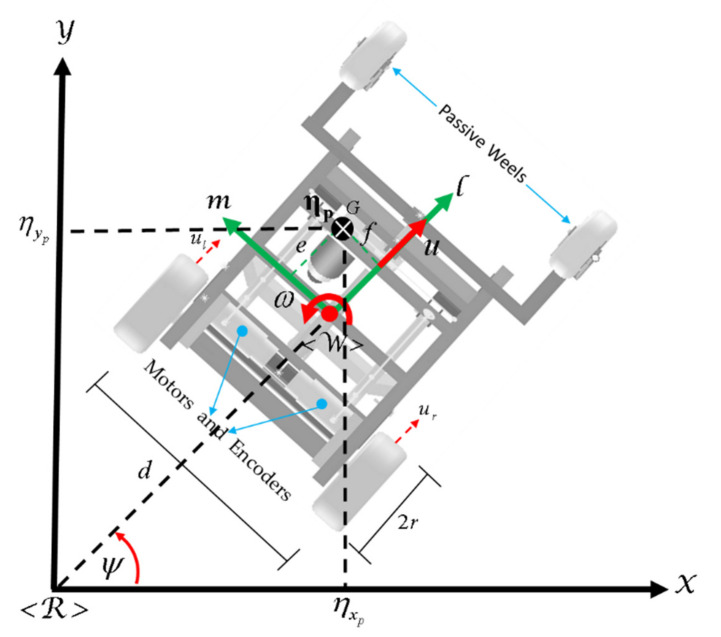
Schematic of the robotic wheelchair.

This work was developed using a human–wheelchair system, consisting of a standing wheelchair robot. The robotic system is a mobile robot consisting of a differential drive, which allows the robot to rotate on the vertical axis freely. The human–wheelchair system moves on a flat horizontal surface, where vertical disturbances are negligible for the system. $R\left(X,Y,Z\right)$ is a fixed reference frame, where the vertical axis is Z, and where it is possible to obtain the motion kinematics of the mobile platform considering the position of the laterally displaced center of mass as a point of interest (see Figure 2).

Therefore, the motion kinematics of the base platform in the X−Y plane can be calculated as:

(6) $$\left[\begin{array}{c} {\dot{\eta }}_{x_{p}}\\ {\dot{\eta }}_{y_{p}} \end{array}\right]=\left[\begin{array}{cc} \cos\psi & -\left(e\sin\psi +f\cos\psi \right)\\ \sin\psi & \left(e\cos\psi -f\sin\psi \right) \end{array}\right]\left[\begin{array}{c} u\\ \omega_{\psi } \end{array}\right]$$

(7) $${\dot{\eta }}_{p}\left(t\right)=J_{p}\left(\psi \right)\mu_{p}\left(t\right)$$

where e and f are distances measured in meters; $J_{p}\left(\psi \right)$ represents the non-holonomic movement configuration of the wheelchair on the X−Y plane, and $\mu_{p}\left(t\right)$ is the wheelchair maneuverability velocity vector; ${\dot{\eta }}_{p}\left(t\right)$ represents the velocity vector of the laterally displaced center of mass of the human–wheelchair system.

To derive the dynamic equations obtained for the human–wheelchair system, the Lagrangian formalism is implemented. In this case, the potential energy $P\left(q\right)=0$, because the trajectory that the standing wheelchair can take is limited to the horizontal plane, i.e., the potential energy is constant because the system has no change in the vertical position. Therefore, the power energy is defined by:

(8) $$K=\frac{1}{2}mv^{2}+\frac{1}{2}I\omega_{\psi }^{2}$$

The total mass of the human–wheelchair system is defined by $m=m_{w}+m_{h}$, where the human mass is $m_{h}$ and the wheelchair mass is $m_{w}$; $v^{2}={\dot{\eta }}_{x_{p}}^{2}+{\dot{\eta }}_{y_{p}}^{2}$ represents the velocity of the wheelchair in the X−Y plane; I is the moment of inertia of the human–wheelchair system, I is around the vertical axis located at G, G being the center of mass of the system in the X−Y plane. Considering that the energy power $P\left(q\right)=0$, we can conclude that L=K.

Now, applying the $\frac{d}{dt}\left(\frac{\partial L}{\partial {\dot{\eta }}_{p}}\right)‒\frac{\partial L}{\partial \eta_{p}}$ with $\eta_{p}=[\begin{array}{ccc} \eta_{x_{p}} & \eta_{y_{p}} & \psi \end{array}]$, it is possible to obtain the dynamic equations of the wheelchair, defined as:

(9) $$\begin{array}{r} \left[\begin{array}{cc} \cos\psi /r\; & \cos\psi /r\;\\ \begin{array}{c} \sin\psi /r\;\\ R/r\; \end{array} & \begin{array}{c} \sin\psi /r\;\\ -R/r\; \end{array} \end{array}\right]\left[\begin{array}{c} \tau_{r}\\ \tau_{l} \end{array}\right]=\left[\begin{array}{ccc} \left(m_{w}+m_{h}\right) & 0 & -\left(m_{w}+m_{h}\right)\left(a\sin\psi +b\cos\psi \right)\\ 0 & \left(m_{w}+m_{h}\right) & \left(m_{w}+m_{h}\right)\left(a\cos\psi -b\sin\psi \right)\\ -\left(m_{w}+m_{h}\right)\left(a\sin\psi +b\cos\psi \right) & \left(m_{w}+m_{h}\right)\left(a\cos\psi -b\sin\psi \right) & I \end{array}\right]\left[\begin{array}{c} {\ddot{\eta }}_{x_{p}}\\ {\ddot{\eta }}_{y_{p}}\\ \ddot{\psi } \end{array}\right]+...\\ \;\left[\begin{array}{ccc} 0 & 0 & \dot{\psi }\left(m_{w}+m_{h}\right)\left(b\sin\psi -a\cos\psi \right)\\ 0 & 0 & -\dot{\psi }\left(m_{w}+m_{h}\right)\left(a\sin\psi b\cos\psi \right)\\ \dot{\psi }\left(m_{w}+m_{h}\right)\left(b\sin\psi -a\cos\psi \right) & -\dot{\psi }\left(m_{w}+m_{h}\right)\left(a\sin\psi b\cos\psi \right) & 0 \end{array}\right]\left[\begin{array}{c} {\dot{\eta }}_{x_{p}}\\ {\dot{\eta }}_{y_{p}}\\ \dot{\psi } \end{array}\right] \end{array}$$

The equation above is expressed in matrix form:

(10) $$B\left(\eta_{p}\right)\tau \left(t\right)={\bar{M}}_{p}\left(\eta_{p}\right){\ddot{\eta }}_{p}\left(t\right)+{\bar{C}}_{p}\left(\eta_{p},{\dot{\eta }}_{p}\right){\dot{\eta }}_{p}\left(t\right)$$

where the inertia of the human–wheelchair system is represented by ${\bar{M}}_{p}\left(\eta_{p}\right)\in R^{nxn}$, which is a positive definite symmetric matrix. The centripetal force matrix of the system is represented by ${\bar{C}}_{p}\left(\eta_{p},{\dot{\eta }}_{p}\right)\in R^{nxn}$; ${\ddot{\eta }}_{p}\left(t\right)$ and ${\dot{\eta }}_{p}\left(t\right)$ are the acceleration and velocity vectors, respectively, with respect to the <R> reference axis, and the input transformation matrix of the system is $B\left(\eta_{p}\right)\in R^{nxr}$. Finally, $\tau \left(t\right)=[\begin{array}{cc} \tau_{r} & \tau_{l} \end{array}]\in R^{2x1}$ is the input vector representing the right and left motor torques, respectively. In order to modify the dynamic model, the linear and angular velocity obtained from the moving platform must be considered as inputs; the following considerations are taken into account:

(11) $${\ddot{\eta }}_{p}\left(t\right)=\frac{d}{dt}{\dot{\eta }}_{p}=J_{p}\left(\psi \right){\dot{\mu }}_{p}\left(t\right)+{\dot{J}}_{p}\left(\psi ,\dot{\psi }\right)\mu_{p}\left(t\right)$$

where:

(12) $${\dot{J}}_{p}\left(\psi ,\dot{\psi }\right)=\left[\begin{array}{cc} \dot{\psi }\sin\psi & \dot{\psi }\left(b\sin\psi -a\cos\psi \right)\\ \begin{array}{c} \dot{\psi }\cos\psi \\ 0 \end{array} & \begin{array}{c} -\dot{\psi }(a\sin\psi +b\cos\psi )\\ 0 \end{array} \end{array}\right]$$

Commercial robots usually integrate PID controllers to compensate for the dynamics of the mechanisms. They take as input linear and angular velocities, and as output, the measurement of them through encoders, leaving aside the electrical part of the motors and the common models that represent the actuators. Therefore, it is necessary to express the dynamic model of the robotic platform considering the longitudinal and rotational velocities through various considerations. To achieve this, these controllers are taken into account and included in the modeling developed in this work. Assuming that the right and left motors have similar characteristics, the actuator models, without considering inductance voltages, according to [17], are expressed in the form

(13) $$\tau_{r}=\frac{k_{a}}{R_{a}}\left(\upsilon_{r}-k_{b}\omega_{r}\right)$$

(14) $$\tau_{l}=\frac{k_{a}}{R_{a}}\left(\upsilon_{l}-k_{b}\omega_{l}\right)$$

The input voltages $\upsilon_{r}$ and $\upsilon_{l}$ are applied to the right and left motor of the standing wheelchair; the angular velocities are $\omega_{r}$ and $\omega_{l}$ for the right and left wheels respectively; $k_{a}$ is the torque constants multiplied by the gear ratio; $k_{b}$ is the electromotor constants multiplied by the gear radio constant; $R_{a}$ is the electric resistance; and finally, $\tau_{r}$ and $\tau_{l}$ are the torques of the left and right motors of the wheelchair multiplied by the gear ratio constant.

Considering that r represents the radius of the left and right wheels and the distance between the wheels is defined by d, the linear and angular velocities u and $\omega_{\psi }$ of the platform without including the slip velocities can be expressed, according to [17]:

(15) $$u=\frac{r}{2}\left(\omega_{r}+\omega_{l}\right)$$

(16) $$\omega_{\psi }=\frac{r}{d}\left(\omega_{r}-\omega_{l}\right)$$

Besides, it is known that:

(17) $$\tau_{u}=\frac{1}{2}\left(\tau_{r}+\tau_{l}\right)$$

(18) $$\tau_{\omega z}=\frac{1}{2}\left(\tau_{r}-\tau_{l}\right)$$

Simple PD servo controllers are considered to control each joint. They are described by the following expressions, according to [17].

(19) $$v_{u}=k_{p}\left(u_{ref}-u\right)+k_{d}\left({\dot{u}}_{ref}-\dot{u}\right)$$

(20) $$v_{\omega_{\psi }}=k_{p}\left(\omega_{\psi }{}_{{}_{ref}}-\omega_{\psi }\right)+k_{d}\left({\dot{\omega }}_{\psi_{ref}}-{\dot{\omega }}_{\psi }\right)$$

where $v_{u}=\frac{1}{2}\left(v_{r}+v_{l}\right)$, $v_{\omega \psi }=\frac{1}{2}\left(v_{r}-v_{l}\right)$. Variables u and $\omega_{\psi }$ represent the longitudinal velocities and rotational velocities of the standing wheelchair. Variables $u_{ref}$ and $\omega_{\psi }{}_{{}_{ref}}$ are the reference velocities of the wheelchair. The variables ${\dot{u}}_{ref}$ and ${\dot{\omega }}_{\psi }{}_{{}_{ref}}$ have been neglected to further simplify the model. The simplifications in the model are valid under the assumption that the servo loops are fast enough. Finally, from Equations (6)–(20), we obtain the dynamic model of the wheelchair; the control signals to be considered are the reference velocities of the wheelchair robot:

(21) $$\left[\begin{array}{c} u_{ref}\\ \omega_{z}{}_{{}_{ref}} \end{array}\right]=\left[\begin{array}{cc} \varsigma_{1_{p}}+\varsigma_{2_{p}}m_{h} & -\left(\varsigma_{3_{p}}+\varsigma_{4_{p}}m_{h}\right)\\ -\left(\varsigma_{5_{p}}+\varsigma_{6_{p}}m_{h}\right) & \varsigma_{7_{p}}+\varsigma_{8_{p}}m_{h} \end{array}\right]\left[\begin{array}{c} \dot{u}\\ {\dot{\omega }}_{z} \end{array}\right]+\left[\begin{array}{cc} \varsigma_{9_{p}} & \dot{\psi }\left(\varsigma_{10_{p}}+\varsigma_{11_{p}}m_{h}\right)\\ 0 & \varsigma_{12_{p}} \end{array}\right]\left[\begin{array}{c} u\\ \omega_{\psi } \end{array}\right]$$

Equation (21) can be compactly described as,

(22) $$\mu_{ref_{p}}\left(t\right)=M_{p}\left(\varsigma \right){\dot{\mu }}_{p}+C_{p}\left(\varsigma ,\mu_{p}\right)\mu_{p}$$

The system’s inertia standing wheelchair robotics are represented by $M_{p}\left(\varsigma \right)\in R^{nxn}$ where n=2; the resulting centripetal forces are represented by $C_{p}\left(\varsigma ,\mu_{p}\right)\in R^{nxn}$; $\mu_{p}\in R^{n}$ and $\mu_{p}=[\begin{array}{cc} u & \omega \end{array}]$ are the robotic standing wheelchair system velocities; $\mu_{ref_{p}}\left(t\right)\in R^{n}$ and $\mu_{ref_{p}}=[\begin{array}{cc} u_{ref} & \omega_{ref} \end{array}]$ are the vector of velocity control signals for the robotic system; finally, the vector of dynamic parameters are represented by $\varsigma_{p}\in R^{l_{p}}$, with $l_{p}=12$ and $\varsigma_{p}=[\begin{array}{cccc} \varsigma_{1_{p}} & \varsigma_{2_{p}} & \ldots & \varsigma_{l_{p}} \end{array}]$ is considered the physical, mechanical, and electrical parameters of the standing wheelchair dynamics. Appendix A shows the dynamic parameters of the wheelchair.

#### 2.2.2. Bipedestation Dynamic Model

**Figure 3:**
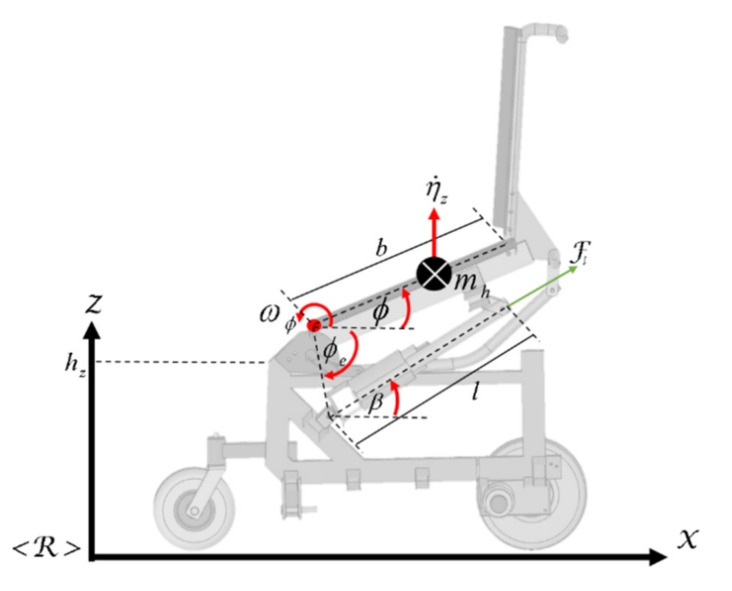
Linear movement of the standing wheelchair on the Z axis.

The dynamics of the bipedestation of the robotic wheelchair are obtained from the linear velocity on the Z axis with respect to the <R> reference system (see Figure 3), where:

(23) $${\dot{\eta }}_{z}\left(t\right)=\omega_{\phi }\left(t\right)b\cos\phi \left(t\right)$$

Now, the angular velocity $\omega_{\phi }\left(t\right)$ can be defined as a nonlinear function, given by:

(24) $$\omega_{\phi }\left(t\right)=\dot{\phi }\left(t\right)=\frac{K_{z}\varphi +K_{l}K_{z}}{K_{h}\sin\left(\phi +\phi_{e}\right)}\dot{\varphi }$$

where ϕ represents the angular position of the inclination of the back of the wheelchair; $\phi_{e}$ is a constant angle of displacement; φ and $\dot{\varphi }$ represent the position and angular velocity, respectively, of the bipedestation motor; and $K_{z}$, $K_{l}$, and $K_{h}$ are distances.

Similar to the previous case, we derive the dynamic equations obtained from the bipedalization of the robotic system using the Lagrangian formalism. In this case, the Lagrangian equation is defined as

(25) $$L=\frac{1}{2}m_{h}{\dot{\eta }}_{z}^{2}-m_{h}g\left(h_{z}+b\sin\left(\phi \right)\right)$$

where $h_{z}$ is the constant height of the wheelchair seat. Now, applying $\frac{d}{dt}\left(\frac{\partial L}{\partial {\dot{\eta }}_{z}}\right)‒\frac{\partial L}{\partial \eta_{z}}$, it is possible to obtain the dynamic equations of the bipedestation, defined as

(26) $$\tau_{\phi }=\left[m_{h}b^{2}\cos^{2}\left(\phi \right)\right]\left[{\dot{\omega }}_{\phi }\right]+\left[-m_{h}b^{2}\omega_{\phi }\cos\left(\phi \right)\sin\left(\phi \right)\right]\left[\omega_{\phi }\right]+\left[m_{h}gb\cos\left(\phi \right)\right]$$

(27) $$\tau_{\phi }\left(t\right)={\bar{M}}_{b}\left(\phi \right){\dot{\omega }}_{\phi }+{\bar{C}}_{b}\left(\phi ,\omega_{\phi }\right)\omega_{\phi }+\bar{g}\left(\phi \right)$$

where ${\bar{M}}_{b}\left(\phi \right)\in R^{+}$ definite the inertia component; ${\bar{C}}_{b}\left(\phi ,\dot{\phi }\right)\in R$ represents the centripetal force component; $\bar{g}\left(\phi \right)$ represents the gravitational component; ${\dot{\omega }}_{\phi }\left(t\right)$ and $\omega_{\phi }\left(t\right)$ represent the angular velocity and acceleration, respectively, of the inclination of the back of the wheelchair, and $\tau_{\phi }\left(t\right)$ represents the torque of the inclination of the back of the wheelchair. In order to consider the dynamic model (18) as velocity inputs, respective conversion must be carried out in Equation (26). The first derivative with respect to time of Equation (24) is obtained, in order to obtain ${\dot{\omega }}_{\phi }$:

(28) $${\dot{\omega }}_{\phi }=\frac{d}{dt}\left(\omega_{\phi }\right)=\frac{K_{z}\varphi +K_{l}K_{z}}{K_{h}\sin\left(\phi +\phi_{e}\right)}\ddot{\varphi }+\frac{K_{h}\sin\left(\phi +\phi_{e}\right)\dot{\varphi }-K_{z}K_{h}\omega_{\phi }\cos\left(\phi +\phi_{e}\right)\left(K_{z}\varphi +K_{l}\right)}{K^{2}{}_{h}\sin^{2}\left(\phi +\phi_{e}\right)}\dot{\varphi }$$

To relate the $\tau_{\phi }$ with the torque of the standing motor $\tau_{\varphi }$, the following nonlinear equation is defined.

(29) $$\tau_{\phi }=T_{a}\cos\left(T_{b}\right)\sin\left(T_{b}\right)\sin\left(\beta -\phi \right)\tau_{\varphi }$$

where $T_{a}$ and $T_{b}$ are positive constants; and β is defined as:

(30) $$\beta =\tan^{-1}\left(\frac{T_{c}\sin\left(\phi -T_{d}\right)+T_{e}}{T_{c}\sin\left(\phi -T_{d}\right)-T_{f}}\right)$$

Now, it is assumed the model of the current motors without considering the inductance voltages are:

(31) $$\tau_{\varphi }=\frac{k_{a_{\varphi }}}{R_{a}{}_{\varphi }}\left(\upsilon_{\dot{\varphi }}-k_{b}\dot{\varphi }\right)$$

where the input voltage to the bipedestation motor is $\upsilon_{\varphi }$; $\dot{\varphi }$ is the angular velocity of the motor; the electromotor constant $k_{b\varphi }$, is multiplied by the gear ratio constant; the electrical resistance is $R_{a\varphi }$; the motor torque multiplied by the gear ratio constant is defined by $\tau_{\varphi }$; and $k_{a\varphi }$ is the torque constant multiplied by the gear ratio. Now, a simple PD servo controller is considered to control bipedestation of the robotic chair, which is described by the following expression:

(32) $$\upsilon_{\dot{\varphi }}=k_{p_{\dot{\varphi }}}\left({\dot{\varphi }}_{{}_{ref}}-\dot{\varphi }\right)+k_{d_{\dot{\varphi }}}\left({\ddot{\varphi }}_{{}_{ref}}-\ddot{\varphi }\right)$$

where ${\ddot{\varphi }}_{{}_{ref}}$ is neglected to further simplify the model. The simplification in the model is valid under the assumption that the servo loop is sufficiently fast. Finally, from Equations (23)–(32), we obtain the dynamic model of the bipedestation of the wheelchair, regarding as control signal the reference angular velocity.

(33) $$\begin{array}{cc} {\dot{\varphi }}_{{}_{ref}}= & \left[\varsigma_{13}+\left(\varsigma_{14}+\varsigma_{15}\varphi \right)\frac{\cos{\left(\phi \right)}^{2}}{\sin\left(\phi +\phi_{e}\right)\sin\left(\beta -\phi \right)}\right]{\dot{\omega }}_{\phi }+\ldots \\ & \left[\varsigma_{16}+\left(\varsigma_{17}\dot{\varphi }\cos{\left(\phi \right)}^{2}+\left(\varsigma_{20}\omega_{\phi }+\varsigma_{21}\varphi \omega_{\phi }\right)\sin\left(2\phi \right)\right)\frac{1}{\sin{\left(\phi +\phi_{e}\right)}^{2}\sin\left(\beta -\phi \right)}+\ldots \right.\\ & \left.\left(\varsigma_{18}\omega_{\phi }+\varsigma_{19}\varphi \omega_{\phi }\right)\frac{\cos{\left(\phi \right)}^{2}\cos\left(\phi +\phi_{e}\right)}{\sin\left(\phi +\phi_{e}\right)\sin\left(\beta -\phi \right)}\right]\omega_{\phi }+\ldots \\ & \left[\varsigma_{22}g\frac{\cos\left(\phi \right)}{\sin\left(\beta -\phi \right)}\right] \end{array}$$

Equation (33) can be compactly described as,

(34) $$\omega_{\phi_{ref}}\left(t\right)=M_{b}\left(\phi ,\varphi \right){\dot{\omega }}_{\phi }+C_{b}\left(\phi ,\dot{\phi },\varphi ,\dot{\varphi }\right)\omega_{\phi }+g\left(\phi \right)$$

The vector of dynamic parameter is defined by $\varsigma_{b}=[\begin{array}{cccc} \varsigma_{1_{b}} & \varsigma_{2_{b}} & \ldots & \varsigma_{l_{b}} \end{array}]\in R^{l_{b}}$ with $l_{b}=10$, containing the physical, mechanical, and electrical parameters of the standing wheelchair dynamics. Appendix B shows the dynamic parameters of the standing wheelchair.

Remark 1: The full mathematical representation of the standing human–wheelchair system based in the instantaneous kinematic model is represented by Equation (4), while the full dynamic model is obtained from Equations (22) and (34). Hence, in (35) is defined the full dynamic model of the standing human–wheelchair,

(35) $$\left[\begin{array}{c} \mu_{ref_{p}}\left(t\right)\\ \omega_{\phi_{ref}}\left(t\right) \end{array}\right]=\left[\begin{array}{c} M_{p}\left(\varsigma \right)\\ M_{b}\left(\phi ,\varphi \right) \end{array}\right]\left[\begin{array}{c} {\dot{\mu }}_{p}\\ {\dot{\omega }}_{\phi } \end{array}\right]+\left[\begin{array}{c} C_{p}\left(\varsigma ,\mu_{p}\right)\\ C_{b}\left(\phi ,\dot{\phi },\varphi ,\dot{\varphi }\right) \end{array}\right]\left[\begin{array}{c} \mu_{p}\\ \omega_{\phi } \end{array}\right]+\left[\begin{array}{c} 0_{2x1}\\ g\left(\phi \right) \end{array}\right]$$

Equation (35) can be compactly described as,

(36) $$\mu_{ref}\left(t\right)=M\left(\phi ,\varphi ,\varsigma \right)\dot{\mu }+C\left(\phi ,\dot{\phi },\varphi ,\varsigma ,\mu \right)\mu +g(\phi )$$

where $M\left(\phi ,\varphi ,\varsigma \right)\in R^{nxn}$ with n=3 represents the inertia matrix of the standing human–wheelchair system; $C\left(\varsigma ,\mu \right)\in R^{nxn}$ represents the centripetal and Coriolis forces; $g\left(\phi \right)\in R^{n}$ represents the gravitational vector; $\mu =[\begin{array}{ccc} u & \omega_{\psi } & \omega_{\phi } \end{array}]\in R^{n}$ are the system velocities; and the vector of velocity control signals is defined by $\mu_{ref}=[\begin{array}{ccc} u_{ref} & \omega_{\psi_{ref}} & \omega_{\varphi_{ref}} \end{array}]\in R^{n}$ for the standing human–wheelchair system; and $\varsigma =[\begin{array}{cc} \varsigma_{p} & \varsigma_{b} \end{array}]\in R^{l}$ with $l=l_{p}+l_{b}=22$ is the vector of dynamic parameters, which contain the physical, mechanical, and electrical parameters of the human–wheelchair system.

From Equation (36) the properties of the proposed dynamic model were obtained:

Property 1. $M\left(\phi ,\varphi ,\varsigma \right)$ is a matrix positive definite, and it is known that $\left\|M\left(\phi ,\varphi ,\varsigma \right)\right\|<k_{M}$.

Property 2. Additionally, also satisfies the following inequalities, $\left\|C\left(\phi ,\dot{\phi },\varphi ,\varsigma ,\mu \right)\right\|<k_{c}\left\|\mu \right\|$.

Property 3. g(ϕ) is vector bounded by, $\left\|g(\phi )\right\|<k_{g}$. where $k_{c}$, $k_{M}$, and $k_{g}$ are positive constants.

Property 4. The dynamic model of the standing human–wheelchair system is represented by

(37) $$M\left(\phi ,\varphi ,\varsigma \right)\dot{\mu }+C\left(\phi ,\dot{\phi },\varphi ,\varsigma ,\mu \right)\mu +g(\phi )=\;\;\Phi \left(\phi ,\dot{\phi },\varphi ,\mu \right)\;\varsigma =\mu_{ref}\left(t\right)$$

where $\Phi \left(\phi ,\dot{\phi },\varphi ,\mu \right)$ $\in R^{nx\;l}$and $\varsigma =[\begin{array}{cccc} \varsigma_{1} & \varsigma_{2} & \ldots & \varsigma_{l} \end{array}]$ are the vector unknown parameters defined by *l* for the human–wheelchair system, i.e., mass of the wheelchair, mass of the human, physical parameters of the wheelchair, motors, velocity, etc.

## 3. Scheme Design Control

**Figure 4:**
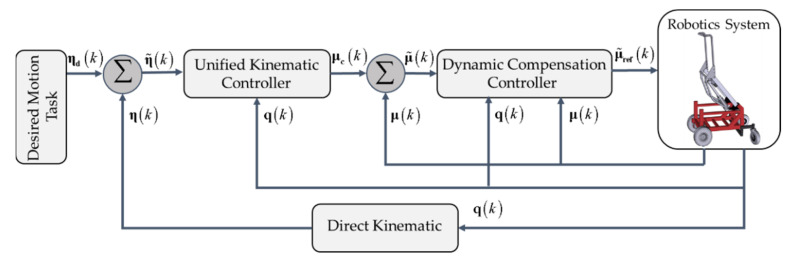
Diagram of the motion control of the standing human–wheelchair system.

The movement control problem of the standing wheelchair is solved with the proposed control scheme shown in Figure 4. This control scheme solves the control problems associated to the route tracking. The design of the controller takes advantage of linear algebra theory and numerical methods, using Euler approximations of the model of the human–wheelchair system. Two cascaded subsystems were implemented for the design of the controller:

(1) Unified kinematic controller, which allows saturation of velocity commands, and its input $\eta_{d}\left(k\right)$ describes the desired motion task of the standing wheelchair, respect to the reference axis $R\left(X,Y,Z\right)$. The control error is defined as $\tilde{\eta }\left(k\right)=\eta_{d}\left(k\right)-\eta \left(k\right)$. Hence, the control object is expressed as

(38) $$\underset{k\to \infty }{\lim}\tilde{\eta }\left(k\right)=0\in R^{m}\;\mathrm{with}\;k\in \left\{1,2,3,4,..\right\}$$

(2) Dynamic compensation controller, whose main objective is to compensate the dynamics of the human–robot system by reducing the velocity tracking error. This controller receives as inputs $\mu_{c}\left(k\right)$, the velocity calculated by the kinematic controller, and obtains velocity references $\mu_{ref}\left(k\right)$ for the robotic standing wheelchair. The velocity control error is defined as $\tilde{\mu }\left(k\right)=\mu_{c}\left(k\right)-\mu \left(k\right)$. Hence, the control aim is to ensure that

(39) $$\underset{k\to \infty }{\lim}\tilde{\mu }\left(k\right)=0\in R^{n}\;\mathrm{with}\;k\in \left\{1,2,3,4,..\right\}$$

The design of the proposed control scheme and the stability analysis of the controllers are presented below. In addition, a robustness analysis for velocity errors is presented, errors that can be generated either by disturbances or by modeling errors. In order to facilitate the design of the proposed controllers, Section 3.1 deals with the design methodology to be considered for the kinematic controller and dynamic compensation, whereas Section 3.2 presents the unified control scheme of the standing wheelchair robot, which describes the type of controller to be used according to the task and desired orientation.

### 3.1. Controller Design Methodology

Through tools based on linear algebra theory and numerical methods, the design of the proposed controllers is obtained in this work. The system of the standing wheelchair is represented through a matrix structure, where theorems and axioms of linear algebra are applied, in order to facilitate the search for the solution of a set of equations. Considering the first-order differential equation,

(40) $$\dot{\xi }\left(t\right)=f\left(\xi \left(t\right),x\left(t\right)\right)$$

where the output of the system is represented by $\dot{\xi }\left(t\right)$ with initial conditions $\xi \left(0\right)=\xi_{0};$ the first derivative of the system with respect to time is defined by $\dot{\xi }\left(t\right)$, and $x\left(t\right)$ is the control action. Additionally, $\xi \left(t\right)$ becomes $\xi \left(t\right)$ considering discrete time with $t=kT_{0}$, where $T_{0}$ represents the proposed sampling time according the Nyquist theorem, and x is the number of samples of the continuous response.

Because the state and the control action on the time instant $t\left(k\right)$ is previously known, the system’s state at instant $t\left(k+1\right)$ can be obtained using Euler’s method, as

(41) $$\frac{1}{T_{0}}\left(\xi \left(k+1\right)-\xi \left(k\right)\right)=f\left(\xi \left(k\right),x\left(k\right)\right)$$

The methodology for the design of controllers is based on matrix equations that represent the behavior of the system. Therefore, a system to be controlled can be presented in a matrix form, as

(42) $$\frac{1}{T_{0}}\left(\xi \left(k+1\right)-\xi \left(k\right)\right)=A\left(k\right)x\left(k\right)$$

where $A\left(k\right)$ contains the characteristics and constraint of the system, respect to the inertial frame $R\left(X,Y,Z\right)$.

**Figure 5:**
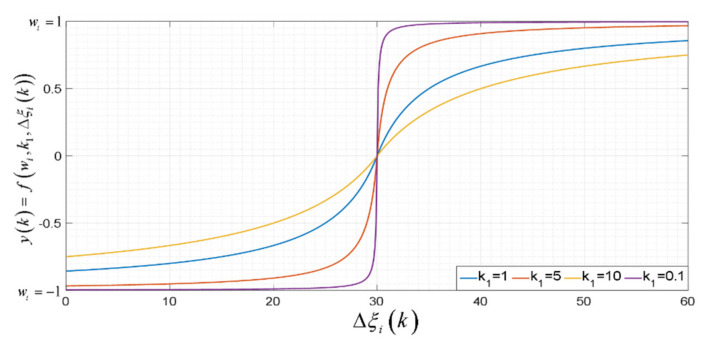
Saturation matrix $W\left(.\right)$ as a function of control error $\Delta \xi \left(k\right)$.

The system evolution using numerical methods is mainly used to determine the system state at instant k+1, if the state and the control action are known at instant k (Markov property). Hence, the desired variable is substituted in variable k+1, to calculate the necessary control action so that the output system changes its current value to a desired one. The following expression is using to achieve the control objective the system:

(43) $$\xi \left(k+1\right)=\xi_{d}\left(k+1\right)-W\left(\Delta \xi \left(k\right)\right)$$

where $\Delta \xi \left(k\right)=\xi_{d}\left(k\right)-\xi \left(k\right)$ represents the variation of control objective; $\xi_{d}\left(k\right)$ is the desired control objective; and W is a diagonal matrix that weighs control errors $\Delta \xi \left(k\right)$

(44) $$W=diag\left(\frac{w_{i}}{k_{1}+\left|\Delta \xi_{i}\left(k\right)\right|}\right)$$

where $k_{1}$ is positive number; $w_{i}$ is a positive number of the diagonal matrix $W\left(.\right)$ weighing the component of the *i*-th control error of the vector $\Delta \xi \left(k\right)$. Equation (44) represents a diagonal matrix that weighs and saturates the control errors, i.e., it has a behavior of a sigmoid function, where $k_{1}$ defines the slope of the saturation function, and $w_{i}$ represents the maximum and minimum saturation value. The behavior of the saturation matrix is shown in Figure 5, for which the saturation function $y\left(k\right)=f\left(w_{i},k_{1},\Delta \xi_{i}\left(k\right)\right)$ is defined as:

(45) $$y\left(k\right)=\left(\frac{w_{i}}{k_{1}+\left|\Delta \xi_{i}\left(k\right)\right|}\right)\Delta \xi_{i}\left(k\right)\;\mathrm{with}\;w_{i}=1\;\mathrm{and}\;\Delta \xi_{i}=[-30:0.1:30]$$

Then, from Equations (41)–(45), the following system of linear equations is determined:

(46) $$\;\underset{A}{\underset{︸}{A\left(k\right)}}\underset{x}{\underset{︸}{x\left(k\right)}}=\underset{b}{\underset{︸}{\frac{1}{T_{0}}\left(\xi_{d}\left(k+1\right)-W\left(\Delta \xi \left(k\right)\right)-\xi \left(k\right)\right)}}$$

The system is rewritten as Ax=b where $A\in R^{mxn}$,$x\in R^{n}$, and $b\in R^{m}$. Namely, $x=A^{-1}b$ defines the control actions, where A is a quadratic matrix, i.e., m=n, and with $\det\left(A\right)\neq 0$; therefore, a direct inverse solution of the matrix A is obtained.

Furthermore, it is said that a system of linear equations is homogeneous if it can be written in the form Ax=0. The configuration of Ax is considered as a redundant system, i.e., the matrix A has more unknowns than equations, where m<n, with rank r=n for each, and taking into account that the homogenous equation has a not-trivial solution, the system could have infinite solutions. In this case, suppose the equation Ax=b is consistent for a given b, and letting $x_{p}\in R^{n}$ be a particular solution, the solution is the set of all the vectors of the form $x=x_{h}+x_{p}$, the homogeneous system Ax=0 yields a solution.

A viable solution to the problem is obtained by defining it as a constrained linear optimization problem $\frac{1}{2}{\left\|x\right\|}_{2}^{2}=\min,$ yielding the particular solution $x_{p}=A^{T}{\left(AA^{T}\right)}^{-1}b$. Additionally, the set of velocities of null space of A in $R^{n}$ do not produce any effect over the actions of the system robotic. It is shown the cost function as $\frac{1}{2}{\left\|x-x_{0}\right\|}_{2}^{2}=\min,$ that yields the homogeneous solution:

(47) $$x_{h}=\left(I_{nxn}-A^{T}{\left(AA^{T}\right)}^{-1}A\right)x_{0}$$

**Figure 6:**
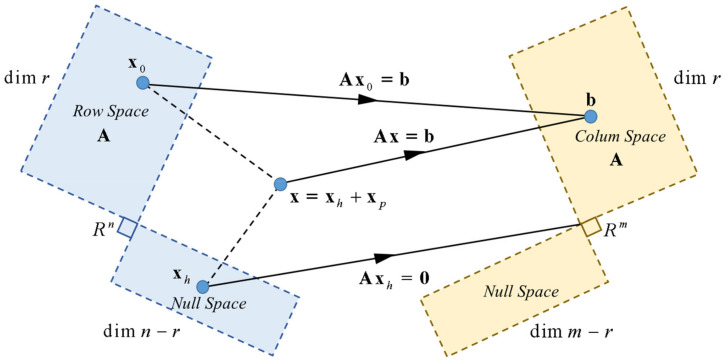
Orthogonal projection of the motion control of the standing wheelchair.

Thus, by manipulating the above equations, the proposed control law is obtained:

(48) $$x=\underset{x_{h}}{\underset{︸}{A^{T}{\left(AA^{T}\right)}^{-1}b}}+\underset{x_{p}}{\underset{︸}{\left(I_{nxn}-A^{T}{\left(AA^{T}\right)}^{-1}A\right)x_{0}}}$$

where a particular solution $x_{p}$ is the first term (left-hand side) and $x_{h}$ is second term of the equation belong to the null space of A. The projection on the null space of the matrix A is represented in Equation (48), where arbitrary vector $x_{0}$ contains the velocities associated with the system robotic, as is shown in Figure 6.

The projection on the null space of the system to be controlled is found in the second term of Equation (48), where the arbitrary vector $x_{0}$ contains the velocities associated with the secondary control objective. The matrix of high-level tasks created by the null space of the system allows to project the velocity of each system in its respective space, where the subtasks compete to solve the problem in different ways. The velocities of the second task are calculated and also included in $x_{0}$, obtaining:

(49) $$x_{0}\left(k\right)=S{\left(k\right)}^{T}{\left(S\left(k\right)S{\left(k\right)}^{T}\right)}^{-1}\frac{1}{T_{0}}\left(\rho_{d}\left(k+1\right)-W_{\rho }\left(\Delta \rho \left(k\right)\right)-\rho \left(k\right)\right)$$

where the Jacobian matrix S contains the secondary objectives of the robotic system to be controlled; Δρ represents the variation of the secondary objective; $\rho_{d}$ is the desired secondary objective; and ρ is the current stat of the secondary objective.

Remark 2: In the robotic standing wheelchair, to include an analytical saturation of velocities, the saturation matrix $W=f\left(w_{ij},k_{1},\Delta \xi_{i}\left(k\right)\right)$ and $W_{\rho }=f\left(w_{\rho ij},k_{\rho 1},\Delta \rho_{i}\left(k\right)\right)$, which limits the control error vectors Δξ and Δρ, are proposed. Thus, Equations (48) and (49) remain bounded, and gains $k_{1}$, $k_{\rho 1}$, $w_{ij}$ and $w_{\rho ij}$ are selected in such a way as to guarantee the control action; $x\left(k\right)$ and $x_{0}\left(k\right)$ remain lower than the maximum admissible velocity values in the standing wheelchair. In the design process of control algorithms for robotic assistance systems focused on the medical area, it is essential that the movements of the robotic systems are limited and smooth, in order to avoid muscle strain to the patient due to poor implementation of control algorithms.

### 3.2. Unified Kinematic Controller

The execution of autonomous tasks of the standing wheelchair robot allows to select the number of outputs to be controlled, and depending on the task to be performed, it can be controlled: (a) displacement in the X‒Y plane without orientation; (b) displacement on the X‒Y plane considering the orientation; (c) displacement in the X‒Y‒Z space without orientation, and (d) displacement in the X‒Y‒Z space considering the orientation. Each of these alternatives must be defined with respect to the reference system $R\left(X,Y,Z\right)$. This subsection describes the control scheme for executing desired tasks that require a control in the X‒Y‒Z space and the desired orientation with respect to the inertial frame $R\left(X,Y,Z\right)$.

Taking into account the kinematic characteristics of the wheelchair, where $J\left(\psi ,\phi \right)\in R^{m\;x\;n}$ with m>n represents the behavior of a sub-powered robotic system; the kinematic controller design was based on the null-space approach, in order to define by separately different tasks and subtasks, unified at the end of the process in order to obtain the control actions of the robotic standing wheelchair. In this work, the main task is that the wheelchair follows a desired path on a planar horizontal surface; and the secondary task is to keep an independent path on the Z axis with an orientation tangential to the path described in the X‒Y plane, with respect to the reference system $R\left(X,Y,Z\right)$.

The Jacobian matrix of the robotic standing wheelchair defined in Equation (4) contains the first derivatives of the system, which correspond to the positions and orientation of the interest point $\dot{\eta }=[\begin{array}{cc} \begin{array}{ccc} {\dot{\eta }}_{x} & {\dot{\eta }}_{y} & {\dot{\eta }}_{z} \end{array} & \dot{\psi } \end{array}]$ with respect to reference system $R\left(X,Y,Z\right)$. In this work, the Jacobian matrix of Equation (50) is considered to calculate different control actions.

(50) $$\left[\begin{array}{c} \left[\begin{array}{c} {\dot{\eta }}_{x}\\ {\dot{\eta }}_{y} \end{array}\right]\\ \left[\begin{array}{c} {\dot{\eta }}_{z}\\ \dot{\psi } \end{array}\right] \end{array}\right]=\left[\begin{array}{c} \left[\begin{array}{ccc} \frac{\partial \eta_{x}}{\partial u} & \frac{\partial \eta_{x}}{\partial \omega_{\psi }} & \frac{\partial \eta_{x}}{\partial \omega_{\phi }}\\ \frac{\partial \eta_{y}}{\partial u} & \frac{\partial \eta_{y}}{\partial \omega_{\psi }} & \frac{\partial \eta_{y}}{\partial \omega_{\phi }} \end{array}\right]\\ \left[\begin{array}{ccc} \frac{\partial \eta_{z}}{\partial u} & \frac{\partial \eta_{z}}{\partial \omega_{\psi }} & \frac{\partial \eta_{z}}{\partial \omega_{\phi }}\\ \frac{\partial \psi }{\partial u} & \frac{\partial \psi }{\partial \omega_{\psi }} & \frac{\partial \psi }{\partial \omega_{\phi }} \end{array}\right] \end{array}\right]\left[\begin{array}{c} u\\ \omega_{\psi }\\ \omega_{\phi } \end{array}\right]$$

Equation (50) can be compactly described as,

(51) $$\left[\begin{array}{c} {\dot{\eta }}_{1}\\ {\dot{\eta }}_{2} \end{array}\right]=\left[\begin{array}{c} J_{1}\\ J_{2} \end{array}\right]\mu$$

The Jacobian matrix represented in Equation (51) is divided into two parts: $J_{1}\left(\psi ,\phi \right)\in R^{2x3}$ are the first derivatives of the interest point positions on a planar horizontal surface, and $J_{2}\left(\psi ,\phi \right)\in R^{2x3}$ are the first derivatives of the interest point on the Z axis and orientation angle; ${\dot{\eta }}_{1}\in R^{2}$ and ${\dot{\eta }}_{2}\in R^{2}$ correspond to the first and second variables to be calculated, respectively. In what follows, the design of control algorithms for the unified kinematic control of the standing wheelchair is presented. It is based on Equation (51) and on the controller design methodology (Section 3.1).

#### 3.2.1. Mobile Platform Controller

To make the point of interest $\eta_{1}\left(\eta_{x},\eta_{y}\right)\in R^{2}$ follow a desired trajectory on a planar horizontal surface, the proposed controller is defined by

(52) $$\mu_{1}\left(k\right)=J_{1}{\left(\psi \left(k\right),\phi \left(k\right)\right)}^{\#}\left(\frac{1}{T_{0}}\left(\eta_{d1}\left(k+1\right)-\eta_{1}\left(k\right)-W_{1}\left(\eta_{d1}\left(k\right)-\eta_{1}\left(k\right)\right)\right)\right)$$

with $J_{1}{\left(\psi \left(k\right),\phi \left(k\right)\right)}^{\#}=J_{1}{\left(\psi \left(k\right),\phi \left(k\right)\right)}^{T}{\left[J_{1}\left(\psi \left(k\right),\phi \left(k\right)\right)J_{1}{\left(\psi \left(k\right),\phi \left(k\right)\right)}^{T}\right]}^{-1}$ representing the right pseudoinverse matrix of $J_{1}\left(\psi \left(k\right),\phi \left(k\right)\right)$. The obtained response in $\mu_{1}\left(k\right)$ are velocities that will be applied to the robotic wheelchair. The velocities are within the point of interest of the desired trajectory on the X‒Y plane; without regard to the bipedestation errors on the Z axis and the desired angle of the wheelchair with respect to reference system $R\left(X,Y,Z\right)$.

#### 3.2.2. Bipedestation and Orientation Control

The second part of the Jacobian matrix (Equation (51) is used for the design of this part of the controller, since it provides the characteristics of bipedestation and orientation $J_{2}\left(\psi ,\phi \right)\in R^{2x3}$. The controller allows the establishment of a secondary objective of the desired bipedestation and orientation $\eta_{2}\left(\eta_{z},\psi \right)\in R^{2}$, defining the controller by:

(53) $$\mu_{2}\left(k\right)=J_{2}{\left(\psi \left(k\right),\phi \left(k\right)\right)}^{\#}\left(\frac{1}{T_{0}}\left(\eta_{d2}\left(k+1\right)-\eta_{2}\left(k\right)-W_{2}\left(\eta_{d2}\left(k\right)-\eta_{2}\left(k\right)\right)\right)\right)$$

Similarly, $J_{2}{\left(\psi \left(k\right),\phi \left(k\right)\right)}^{\#}$ represents the inverse of $J_{2}\left(\psi \left(k\right),\phi \left(k\right)\right)$.

#### 3.2.3. Unified Kinematic Controller

This subsection defines the trajectory tracking control of the interest point on the X‒Y plane. Hence, the bipedestation and orientation control are tasks that do not conflict with the main control objective. The proposed final controller is based on Equation (46), and is presented as:

(54) $$\begin{array}{l} \mu_{c}\left(k\right)=J_{1}{\left(\psi \left(k\right),\phi \left(k\right)\right)}^{\#}\left(\frac{1}{T_{0}}\left(\eta_{d1}\left(k+1\right)-\eta_{1}\left(k\right)-W_{1}\left(\eta_{d1}\left(k\right)-\eta_{1}\left(k\right)\right)\right)\right)+\ldots \\ \left(I_{3x3}-J_{1}{\left(\psi \left(k\right),\phi \left(k\right)\right)}^{\#}J_{1}\left(\psi \left(k\right),\phi \left(k\right)\right)\right)J_{2}{\left(\psi \left(k\right),\phi \left(k\right)\right)}^{\#}\left(\frac{1}{T_{0}}\left(\eta_{d2}\left(k+1\right)-\eta_{2}\left(k\right)-W_{2}\left(\eta_{d2}\left(k\right)-\eta_{2}\left(k\right)\right)\right)\right) \end{array}$$

Equation (54) rewritten in simplified form is

(55) $$\mu_{c}\left(k\right)=\mu_{1}\left(k\right)+\left(I_{3x3}-J_{1}{\left(\psi \left(k\right),\phi \left(k\right)\right)}^{\#}J_{1}\left(\psi \left(k\right),\phi \left(k\right)\right)\right)\mu_{2}\left(k\right)$$

Equation (55) is subdivided into two terms: the first term on the right side describes the primary task of robotic standing wheelchair. Self-motion of the bipedestation is defined in the second term. The matrix $\left(I_{3x3}-J_{1}{\left(\psi \left(k\right),\phi \left(k\right)\right)}^{\#}J_{1}\left(\psi \left(k\right),\phi \left(k\right)\right)\right)$ is projected onto vector $\mu_{2}\left(k\right)$, where the null space of the standing wheelchair Jacobian $N\left(J_{1}\left(\psi \left(k\right),\phi \left(k\right)\right)\right)$ ensures that the main task is not affected by secondary control objectives.

### 3.3. Kinematic Stability Analysis

Taking into account the desired velocity tracking, $\mu_{c}\left(k\right)\equiv \mu \left(k\right)$, the stability analysis is carried out to control the main objective; considering $J_{1}\left(\psi ,\phi \right)J_{1}{\left(\psi \left(k\right),\phi \left(k\right)\right)}^{\#}=I_{2x2}$ in Equation (55), it is multiplied by $J_{1}\left(\psi \left(k\right),\phi \left(k\right)\right)$, obtaining the closed loop equation shown below.

(56) $$\begin{array}{cc} \frac{1}{T_{0}}\left(\eta_{1}\left(k+1\right)-\eta_{1}\left(k\right)\right) & =J_{1}\left(\psi \left(k\right),\phi \left(k\right)\right)\mu_{c}\left(k\right)\\ & =\left(\frac{1}{T_{0}}\left(\eta_{d1}\left(k+1\right)-\eta_{1}\left(k\right)-W_{1}\left(\eta_{d1}\left(k\right)-\eta_{1}\left(k\right)\right)\right)\right) \end{array}$$

(57) $$\eta_{1}\left(k+1\right)-\eta_{1}\left(k\right)=\eta_{d1}\left(k+1\right)-\eta_{1}\left(k\right)-W_{1}\left(\eta_{d1}\left(k\right)-\eta_{1}\left(k\right)\right)$$

Simplifying and considering as ${\tilde{\eta }}_{1}\left(k\right)=\eta_{d1}\left(k\right)-\eta_{1}\left(k\right)$ the control error of the primary objective, such that

(58) $${\tilde{\eta }}_{1}\left(k+1\right)=W_{1}\left({\tilde{\eta }}_{1}\left(k\right)\right)$$

(59) $$\left[\begin{array}{c} {\tilde{\eta }}_{x}\left(k+1\right)\\ {\tilde{\eta }}_{y}\left(k+1\right) \end{array}\right]=\left[\begin{array}{c} w_{x}{\tilde{\eta }}_{x}\left(k\right)\\ w_{y}{\tilde{\eta }}_{y}\left(k\right) \end{array}\right]$$

Table 1 shows the evolution of the *i*-th control error from Equation (59).

If n→∞, the ${\tilde{\eta }}_{x}$ control error at an instant of infinite time will be equal to ${\tilde{\eta }}_{x}\left(\infty \right)=w_{x}^{\infty }{\tilde{\eta }}_{x}\left(1\right)$; hence, if $0<w_{x}<1$ the control error ${\tilde{\eta }}_{x}\left(\infty \right)=0$ with k→∞. Performing an analysis similar to that of the ${\tilde{\eta }}_{x}$ control error, it can be concluded that $\underset{k\to \infty }{\lim}\tilde{\eta }\left(kT_{0}\right)=0$ with $0<\mathrm{diag}\left(W_{1}\right)<1$ when k→∞. In other words, it is asymptotically stable.

### 3.4. Dynamic Compensation Controller

The main objective of the dynamic compensation controller is to reduce the velocity tracking error of the robotic system. This action is carried out by compensating the dynamics of the standing human–wheelchair system. The desired velocity $\mu_{c}\left(k\right)$ is calculated by means of the unified kinematic controller. These velocities are used as inputs of the described controller and the velocity references $\mu_{ref}\left(k\right)$ are generated for the standing wheelchair system (see Figure 4). In case of not having a perfect velocity tracking, the velocity error is defined as $\tilde{\mu }\left(k\right)=\mu_{c}\left(k\right)-\mu \left(k\right)$. Without including perturbations (Equation (36)), the exact model of the standing wheelchair system is considered to be:

(60) $$\mu_{ref}\left(t\right)=M\left(\phi ,\varphi ,\varsigma \right)\dot{\mu }\left(t\right)+C\left(\phi ,\dot{\phi },\varphi ,\varsigma ,\mu \right)\mu \left(t\right)+g(\phi )$$

The design of the dynamic compensation controller is based on the theory of linear algebra and numerical methods. In this sense, the state and the control action of the system at the instant of time $t\left(k\right)$ are known values, which allows us to approximate the value of the state of the system in time $t\left(k+1\right)$ using Euler’s method as

(61) $$\dot{\mu }\left(k\right)=\frac{1}{T_{0}}\left(\mu \left(k+1\right)-\mu \left(k\right)\right)$$

Considering the discretized acceleration in Equation (61) and the discrete dynamic model in Equation (60), we have:

(62) $$\mu_{ref}\left(k\right)=M\left(\phi \left(k\right),\varphi \left(k\right),\varsigma \right)\left(\frac{1}{T_{0}}\left(\mu \left(k+1\right)-\mu \left(k\right)\right)\right)+C\left(\phi ,\dot{\phi },\varphi ,\varsigma ,\mu \right)\mu \left(k\right)+g\left(\phi \left(k\right)\right)$$

Now, applying the Markov property and control errors, the velocity of the standing wheelchair system at instant $t\left(k+1\right)$ can be determined as

(63) $$\mu \left(k+1\right)=\mu_{c}\left(k+1\right)-W_{\mu }\tilde{\mu }\left(k\right)$$

where $\mu \left(k+1\right)$ represents the desired velocities of the robotic system. Then control law is proposed,

(64) $$\mu_{ref}\left(k\right)=M\left(\phi ,\varphi ,\varsigma \right)\left(\frac{1}{T_{0}}\left(\mu_{c}\left(k+1\right)-W_{\mu }\tilde{\mu }\left(k\right)-\mu \left(k\right)\right)\right)+C\left(\phi ,\dot{\phi },\varphi ,\varsigma ,\mu \right)\mu \left(k\right)+g\left(\phi \right)$$

where $W_{\mu }=f\left(w_{\mu_{i}},k_{\mu },{\tilde{\mu }}_{i}\left(k\right)\right)\in R^{3x3}$ is defined similarly to Equation (44); this implies that $W_{\mu }$ represents a diagonal matrix that weighs and limits velocities of the velocity errors vector.

### 3.5. Dynamic Stability Analysis

Ceasing to consider a perfect velocity, i.e., $\mu_{ref}\left(k\right)\neq \mu \left(k\right)$, this subsection discusses the stability of the proposed dynamic compensation controller. Thus, the dynamic model of the robotic system (Equation (41)) is equated with the proposed control law (Equation (46)). The dynamic closed-loop equation is defined by:

(65) $$\begin{array}{l} M\left(\phi \left(k\right),\varphi \left(k\right),\varsigma \right)\left(\frac{1}{T_{0}}\left(\mu \left(k+1\right)-\mu \left(k\right)\right)\right)+C\left(\phi ,\dot{\phi },\varphi ,\varsigma ,\mu \right)\mu \left(k\right)+g\left(\phi \left(k\right)\right)=\ldots \\ M\left(\phi ,\varphi ,\varsigma \right)\left(\frac{1}{T_{0}}\left(\mu_{c}\left(k+1\right)-W_{\mu }\tilde{\mu }\left(k\right)-\mu \left(k\right)\right)\right)+C\left(\phi ,\dot{\phi },\varphi ,\varsigma ,\mu \right)\mu \left(k\right)+g\left(\phi \right) \end{array}$$

Simplifying Equation (65) we have

(66) $$\tilde{\mu }\left(k+1\right)=W_{\mu }\tilde{\mu }\left(k\right)$$

The analysis of the evolution of velocity errors is carried out in a similar way to Equation (59), concluding that if k→∞, the *i-th* velocity error ${\tilde{\mu }}_{i}$ at an instant of infinite time will be equal to ${\tilde{\mu }}_{i}\left(\infty \right)=w_{i}^{\infty }{\tilde{\mu }}_{i}\left(1\right)$. Hence, if $0<w_{i}<1$ the control error ${\tilde{\mu }}_{i}\left(\infty \right)=0$ with k→∞. Hence, it can be concluded that $\underset{k\to \infty }{\lim}\mu \left(kT_{0}\right)=0$ with $0<diag\left(W_{\mu }\right)<1$ when k→∞, therefore, it is asymptotically stable.

### 3.6. Control Scheme Robustness Analysis

This subsection analyzes the evolution of the kinematic controller error $\tilde{\eta }\left(k\right)$. For this purpose, the existence of velocity errors $\tilde{\mu }\left(k\right)$ is considered. Velocity errors can be caused by disturbances in the system and model errors, among others. Hence, Equation (39) can be defined as:

(67) $$\eta_{1}\left(k+1\right)-\eta_{1}\left(k\right)=\eta_{d1}\left(k+1\right)-\eta_{1}\left(k\right)-W_{1}\left(\eta_{d1}\left(k\right)-\eta_{1}\left(k\right)\right)-T_{0}J_{1}\left(\psi \left(k\right),\phi \left(k\right)\right)\tilde{\mu }\left(k\right)$$

(68) $${\tilde{\eta }}_{1}\left(k+1\right)=W_{1}\left({\tilde{\eta }}_{1}\left(k\right)\right)-T_{0}J_{1}\left(\psi \left(k\right),\phi \left(k\right)\right)\tilde{\mu }\left(k\right)$$

Performing an analysis similar to the previous case, it can be concluded that if k→∞ then $\underset{k\to \infty }{\lim}\tilde{\eta }\left(k\right)=$$T_{0}J_{1}\left(\psi \left(k\right),\phi \left(k\right)\right)\tilde{\mu }\left(k\right)$, which implies that the error $\tilde{\eta }\left(kT_{0}\right)$ is delimited by:

(69) $$\left\|\tilde{\eta }\left(kT_{0}\right)\right\|\leq T_{0}\left\|J_{1}\left(\psi \left(k\right),\phi \left(k\right)\right)\tilde{\mu }\left(k\right)\right\|$$

Now, considering that $\psi \left(k\right)$ and $\phi \left(k\right)$ are bounded angles, it is possible to state that $\left\|J_{1}\left(\psi \left(k\right),\phi \left(k\right)\right)\right\|<k_{\psi ,\phi }$. Then, Equation (69) can be expressed as:

(70) $$\left\|\tilde{\eta }\left(kT_{0}\right)\right\|\leq T_{0}k_{\psi ,\phi }\left\|\tilde{\mu }\left(k\right)\right\|$$

The velocity error $\tilde{\mu }\left(k\right)$ can be generated by either obtaining the dynamic model of the robotic system in an incorrect way, or by errors made in the process of identifying the dynamic parameters, or, when performing movement tests with the standing chair, a person with a different weight than the one used in the process of identifying the dynamic parameters. However, if the velocity error is bounded ($\left\|\tilde{\mu }\left(k\right)\right\|\leq k_{\tilde{\mu }}$), then the control error $\left\|\tilde{\eta }\left(kT_{0}\right)\right\|$ is eventually delimited by Equation (70).

## 4. Experimental Results

Experimental results are described in three subsections. The first one deals with the mechanical–electronic construction of the robotic standing wheelchair and presents a brief description of the prototype created in order to run experimental tests. Secondly, we validated the dynamic model of the human–robot system, through experimental identification and validation tests. Thirdly, the validation of the proposed control scheme is carried out through experimental tests supported by graphs showing the effectiveness of the control scheme.

### 4.1. Robotic Standing Wheelchair Construction

**Figure 7:**
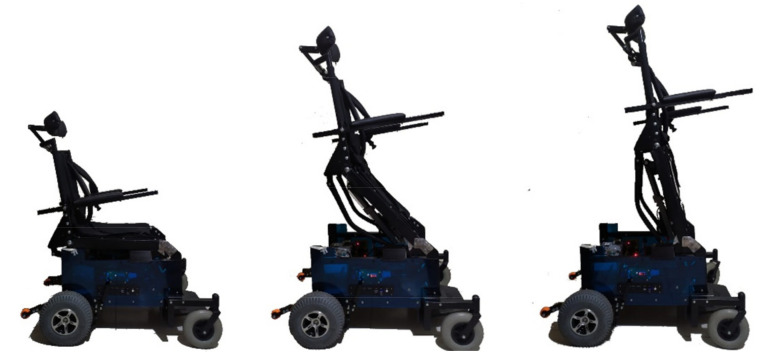
Standing wheelchair at different positions.

This work uses a non-holonomic robotic standing wheelchair, which was developed by the ARSI Research Group of the University of the Armed Forces ESPE. The standing wheelchair has two wheels driven by two DC motors independently (rear traction), in order to move the chair on a planar horizontal surface considering the non-holonomic restriction (Equation (5)). Two passive wheels (pivoting wheels) are located in the front part of the central axis to give greater stability to the robotic standing wheelchair; and an independent linear drive that allows the change from sitting position to standing position, by means of a DC motor. The position and relative orientation of the standing wheelchair can be known by means of the encoders installed on each of the motor shafts. The construction of the mechanical parts of the robotic system have been designed to fit together, resulting in an appropriate analysis of the center of mass and weight distribution, allowing the mobile platform and the standing joint to be unified as a single system as shown in Figure 7.

**Figure 8:**
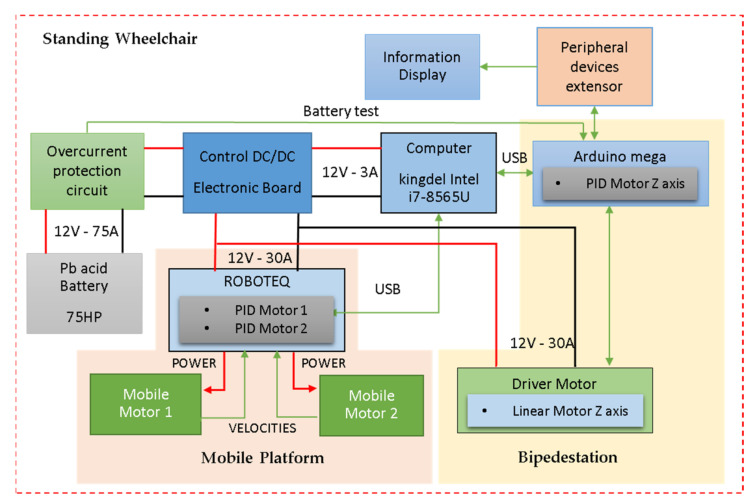
Design elements of the robotic standing wheelchair.

The standing wheelchair robotic system was designed in such a way that the electronic components can be interconnected between the control elements and equipment, power, and energy supply. The system consists of DC motors, overcurrent prevention elements, an electronic control board, a computer, a peripheral extender, and a battery. The distribution of the elements, together with the communication links among them, is shown in Figure 8. We describe below the different parts of the whole system.

**Figure 9:**
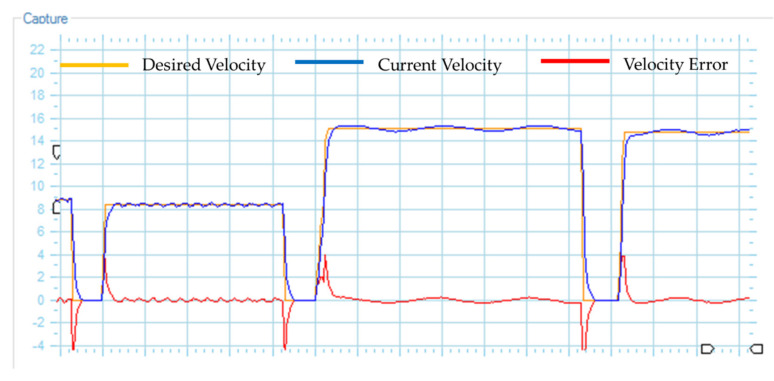
Right motor velocity of the standing wheelchair with a Proportional Integral Derivative controller.

*(i) Mobile platform system:* this section consists of two direct current motor that are controlled by a Roboteq card (Roboteq Inc., Scottsdale, AZ, USA), which incorporates PID controllers through a refeed with encoders of 400 pulses per revolution (velocities are subsequently transformed to rad/s). The motor controller card, through Rs232 serial communication, sends the actual velocity and position of the mobile platform to a computer, whereas the computer sends the maneuverability velocities for the control of the mobile platform (maneuverability velocities obtained by implementing a control algorithm). The PID control implemented in one of the motors is shown in Figure 9, where it is observed that the velocity error tends to zero asymptotically. The Haalman method was considered for the tuning of the PID controllers, and the following parameters were obtained:$k_{p}=1.097$, $k_{i}=0.645$ and $k_{d}=0.02$.

*(ii) the standing system* has an Atmel microcontroller (Atmel Corporation, San José, CA, USA) and a non-commercial control board as a processor, which was designed to satisfy the communication and processing requirements for the correct operation of the standing axis. The standing section consists of a direct current motor. The motor incorporates mechanical velocity reducers and a ball screw coupling, in order to generate a linear movement that allows the wheelchair to move on the Z axis. In addition, the motor has an encoder attached to implement an internal loop PID controller. On the other hand, the standing motor control card (through RS232 serial communication) sends the standing angle and the motor velocity to the computer. On the other hand, the computer sends the maneuverability velocity commands for the standing control of the wheelchair (maneuverability velocity obtained by implementing a control algorithm); *(iii) the computer* has enough resources to process high-level programs. In the computer, the necessary calculations for the implementation of the control algorithms are carried out through the Windows operating system using mathematical software; *(iv) electronic control board*: its function is to distribute and regulate the power voltage of the motors and the computer system. In addition, it consists of voltage and current measurement elements in order to emit warning signals in the event of possible failures, discharges or disconnection of devices. A charging and connection status screen is integrated into the dash; (*v) peripheral ports,* which are responsible for communicating external devices (cameras, memory cards, etc.) with the internal computer; finally, vi) the battery supplies the necessary power to the system, which delivers up to 75 [A/h] with 12 V.

### 4.2. Dynamic Model Identification and Validation

The identification and validation of the dynamic parameters of the mathematical model (Equation (36) that represents the dynamics of the standing human–wheelchair system is tested in this subsection. The main objective of this process is to experimentally determine the numerical values of $\varsigma =[\begin{array}{cccc} \varsigma_{1} & \varsigma_{2} & \ldots & \varsigma_{l} \end{array}]$ with l=22, so that the dynamic model can be used in advanced control algorithms. The identification of dynamic parameters is the way to establish a relationship between the real results and the mathematical model developed, allowing to refine the model obtained until the behavior of the chair–user system shows sufficient precision to meet the requirements of the objectives of the desired control [17,20,34].

In order to identify the dynamic parameters of the human–wheelchair system, Property 4 of the dynamic model described in Section 2.2.1 is considered. For this, in order to estimate the acceleration of the robotic system, a first order filter is applied to Equation (37), obtaining:

(71) $$\;\;\frac{\lambda }{s+\lambda }\Phi \left(\phi ,\dot{\phi },\varphi ,\mu \right)\;\varsigma =\frac{\lambda }{s+\lambda }\mu_{ref}\left(t\right)$$

Rewriting Equation (71) in a compact form, we obtain that:

(72) $$\;\Phi_{F}\left(\phi ,\dot{\phi },\varphi ,\mu \right)\;\varsigma =\mu_{ref\_F}\left(t\right)$$

where  s is the Laplace transform variable, and λ represents a positive fit constant. To estimate the parameters that best fit the dynamic model of the human–wheelchair system, the method of least squares is implemented. Therefore, the following expression is considered,

(73) $$\;\varsigma ={\left(\Phi_{FT}^{T}\Phi_{FT}\right)}^{-1}\Phi_{FT}^{T}\mu_{ref\_FT}$$

where ς represents the values of the calculated dynamic parameters; $\;\Phi_{FT}$ is a matrix that considers the variation of the dynamic model at any instant of time in which the dynamics of the real robotic system was excited; and $\;\mu_{ref\_FT}$ is the vector that incorporates the input excitation signals of the real robotic system. Table 2 shows the dynamic parameters of the standing human–wheelchair system, considering a person of 75 kg mounted on the wheelchair and moving on a wooden surface.

Remark 3: The dynamic parameters presented in Table 2 may vary according to the weight of the person and the type of surface on which the standing wheelchair moves.

**Figure 10:**
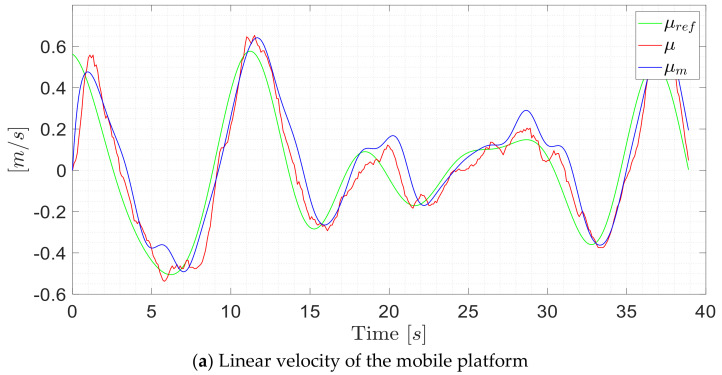

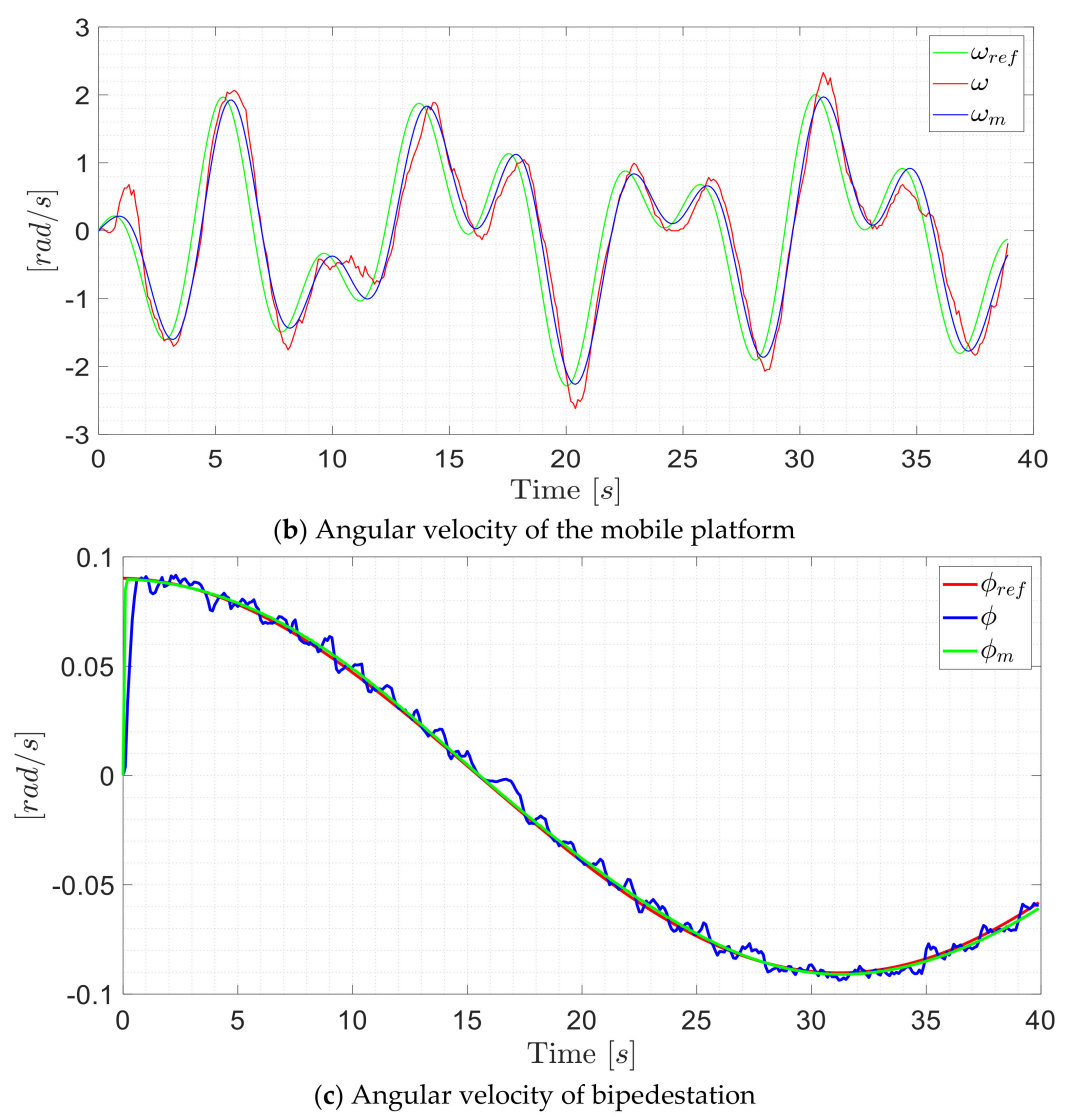
Validation data of the proposed dynamic model of the standing human–wheelchair. The subscript *ref* represents the excitation velocity to the robotic system; *r* represents the current velocity of the system; and *m* is the simulation velocity of the mathematical dynamic model.

Figure 10 presents experimental data for the validation process, where it can be observed the adequacy of the proposed dynamic model.

**Figure 11:**
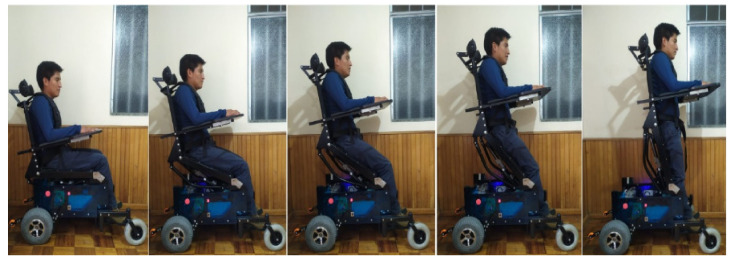
Autonomous movement of the standing human–wheelchair system.

The so-built standing wheelchair allows the user to raise the chair from a seated to a standing position. The mechanism to raise the chair is controlled by the control scheme proposed in this work. Figure 11 shows the autonomous movement of the robotic standing wheelchair with a user of 75 kg.

### 4.3. Control Scheme Implementation

In order to obtain experimental results with the human–wheelchair system for the execution of autonomous tasks, a partially structured scenario was considered. All the experimental tests presented in this work use the wheelchair presented in the Section 4.1. The robotic wheelchair considers linear velocity and angular velocity for mobile platforms as input signals. In addition, it has an angular velocity as input signal for standing control on the Z axis. On the other hand, the standing wheelchair has as output signals the displacement and rotation $\eta \left(\eta_{x},\eta_{y},\eta_{z},\eta_{\psi }\right)\in R^{4}$with relation to reference frame $R\left(X,Y,Z\right)$. In addition, the output signals for the mobile platform were linear and angular velocities, whereas the output signal for the standing position was the angular velocity.

Several experiments on the motion control of the standing wheelchair system were performed in order to illustrate the performance of the proposed controller. The most representative results are presented in the next section. Each one of the experiments was executed with different control objectives. It should be clarified that all experiments were implemented considering the proposed control scheme in Figure 4. The difference of the experiments is in the control law to be implemented in the kinematic controller; the control law is selected based on the desired task.

The parameters of the proposed control scheme were adjusted, as shown in Table 3, for all the experiments. The sampling time was set to $T_{0}=0.1\;[s]$.

(a) First experiment

We consider Equations (52) and (64) for the implementation of the kinematic control law. Equation (52) considers as desired values $\eta_{1d}\left(\eta_{dx},\eta_{dy}\right)\in R^{2}$. Therefore, the desired task of the human–robot system must be defined on the X−Y plane (without considering the orientation) with respect to reference frame <R>. The desired task and initial conditions for the controller are defined in Table 4 for the experiment.

**Figure 12:**
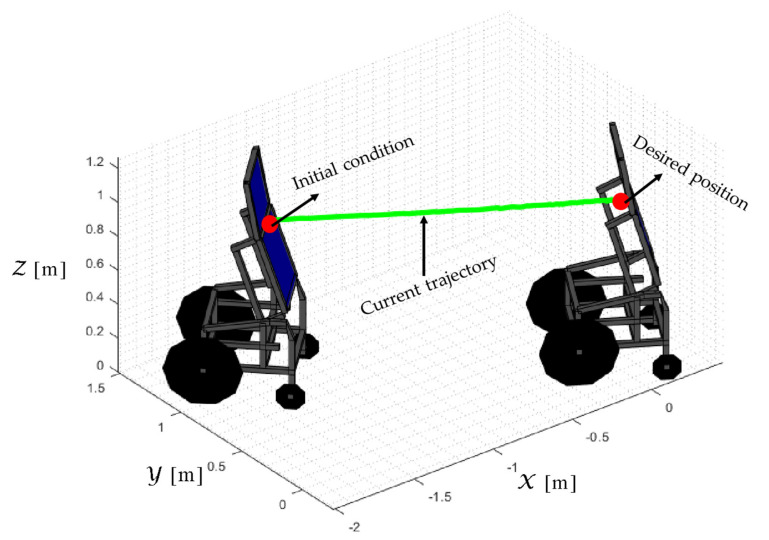
Stroboscopic movement of the human–robot system based on the real experimental data.

**Figure 13:**
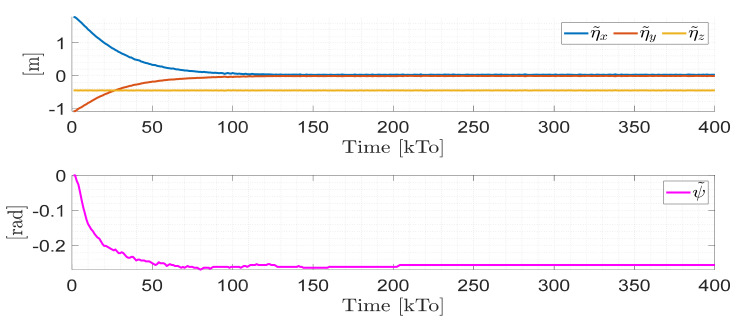
Time evolution of the control errors $\tilde{\eta }\left(kT_{0}\right)=\left({\tilde{\eta }}_{x},{\tilde{\eta }}_{y},{\tilde{\eta }}_{z},\tilde{\psi }\right)$.

**Figure 14:**
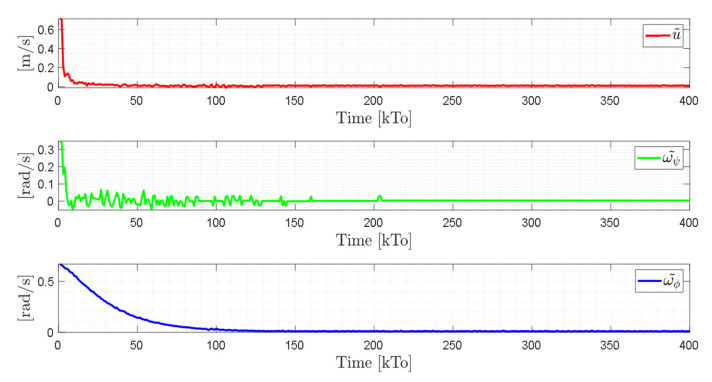
Time evolution of the control errors $\tilde{\mu }\left(kT_{0}\right)=\left(\tilde{u},{\tilde{\omega }}_{\psi },{\tilde{\omega }}_{\phi }\right)$.

**Figure 15:**
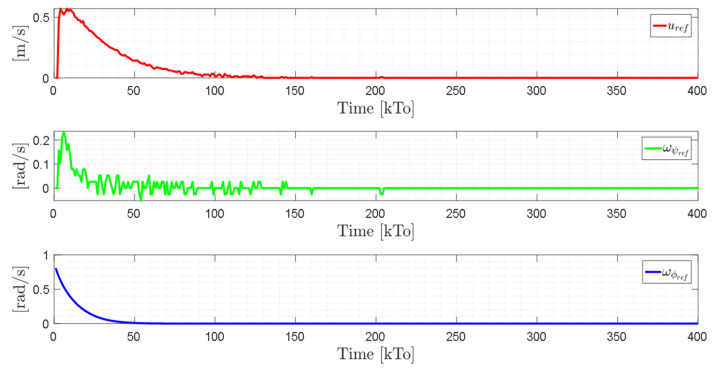
Velocity commands to the standing wheelchair $\mu_{ref}\left(kT_{0}\right)=\left(u_{ref},\omega_{\psi_{ref}},\omega_{\phi_{ref}}\right)$.

The main results of the first experiment are illustrated in Figure 12, Figure 13, Figure 14 and Figure 15. Figure 12 shows the stroboscopic movement of the standing human–wheelchair system, based on real data. Figure 13 and Figure 14 show that the control errors ${\tilde{\eta }}_{1}\left({\tilde{\eta }}_{x},{\tilde{\eta }}_{y}\right)\in R^{2}$ and $\tilde{\mu }\left(\tilde{u},{\tilde{\omega }}_{\psi },{\tilde{\omega }}_{\phi }\right)\in R^{3}$, respectively, converge to values close to zero asymptotically. It should be noted that the kinematic controller fulfills the objective of the desired task, while the dynamic compensation controller compensates for the dynamics of the human–wheelchair system. In other words, they are two independent controllers with different control objectives. The control action of the standing wheelchair is shown in Figure 15.

Control errors ${\tilde{\eta }}_{z}$ and $\tilde{\psi }$ do not tend to zero, because these control states are not part of the desired task, therefore they are not considered in the proposed control law of the Equation (52).

(b) Second experiment

We consider for this experiment the implementation of the kinematic control law based on Equations (53) and (64), respectively. Equation (53) considers as desired values $\eta_{2d}\left(\eta_{dz},\eta_{d\psi }\right)\in R^{2}$, therefore, the desired task of the human–robot system must be defined on the Z axis, considering the orientation respect to inertial reference frame <R>. The desired task and initial conditions for the controller are defined in Table 5.

**Figure 16:**
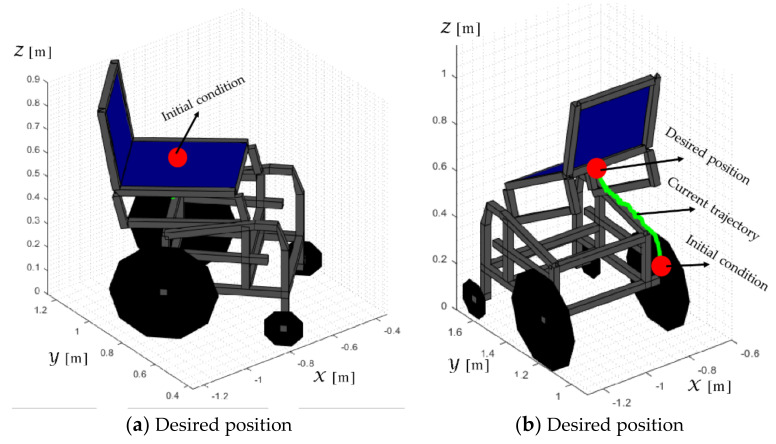
Stroboscopic movement of the human–robot system based on the real experimental data.

**Figure 17:**
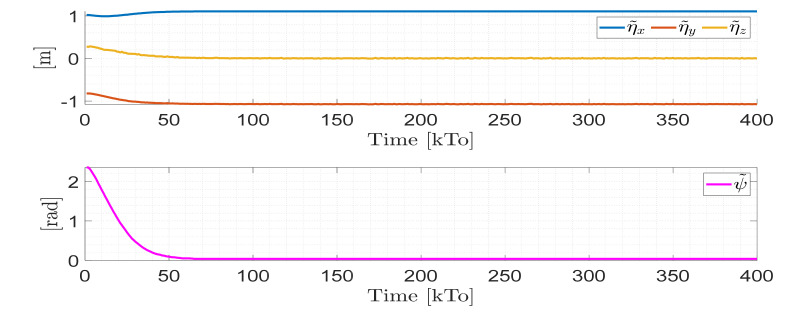
Time evolution of the control errors $\tilde{\eta }\left(kT_{0}\right)=\left({\tilde{\eta }}_{x},{\tilde{\eta }}_{y},{\tilde{\eta }}_{z},\tilde{\psi }\right)$.

**Figure 18:**
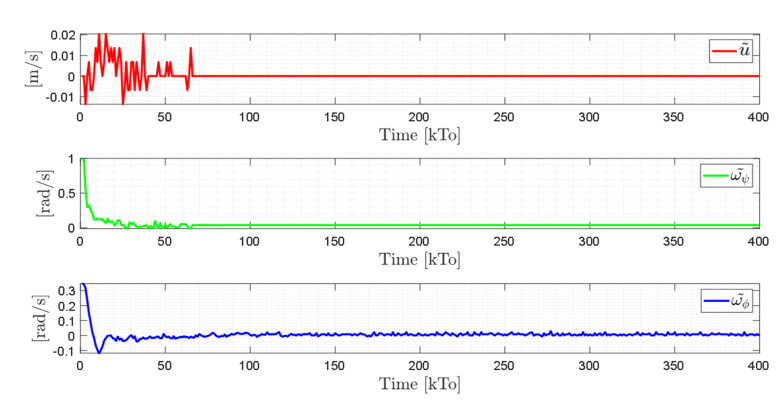
Time evolution of the control errors $\tilde{\mu }\left(kT_{0}\right)=\left(\tilde{u},{\tilde{\omega }}_{\psi },{\tilde{\omega }}_{\phi }\right)$.

**Figure 19:**
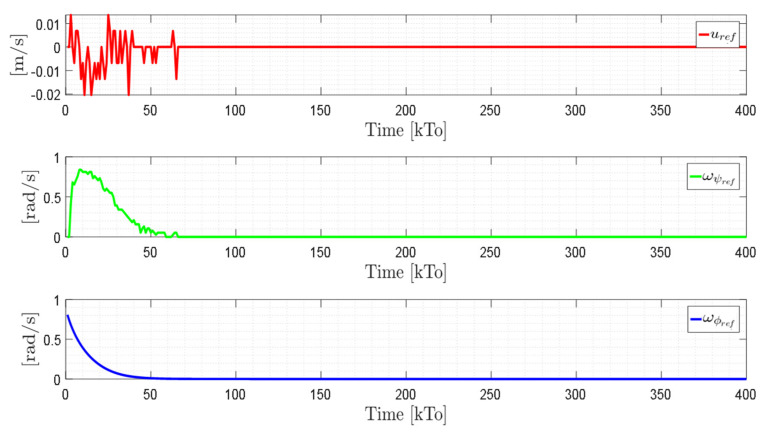
Velocity commands to the standing wheelchair $\mu_{ref}\left(kT_{0}\right)=\left(u_{ref},\omega_{\psi_{ref}},\omega_{\phi_{ref}}\right)$.

The desired task considers a stabilization point on the Z axis and the desired orientation with respect to the X axis of the inertial frame <R>. The main results of the second experiment are shown in Figure 16, Figure 17, Figure 18 and Figure 19. Figure 16 shows the stroboscopic movement of the standing human–wheelchair system, based on real data. Figure 16a shows the standing wheelchair robot in the initial condition, whereas Figure 16b shows the standing wheelchair robot in the desired position. Figure 17 and Figure 18 show that the control errors ${\tilde{\eta }}_{2}\left({\tilde{\eta }}_{z},{\tilde{\eta }}_{\psi }\right)\in R^{2}$and $\tilde{\mu }\left(\tilde{u},{\tilde{\omega }}_{\psi },{\tilde{\omega }}_{\phi }\right)\in R^{3}$, respectively, converge to values close to zero asymptotically. Figure 19 depicts the control actions of the standing wheelchair robot.

Control errors ${\tilde{\eta }}_{x}$ and ${\tilde{\eta }}_{y}$ do not tend to zero, because these control states are not part of the desired task, therefore they are not considered in the proposed control law of the Equation (53).

In previous experiments, the performance of the mobile platform controller expressed in the Equation (52) and the orientation and standing controller expressed in the Equation (53) were tested. Both controllers considered only two of the four desired states that a complex task may require.

(c) Third experiment

For these final experiments, the implementation of the kinematic control law is considered (Equations (55) and (64)). Equation (55) considers a unified control based on primary and secondary objectives. The main objectives considered $\eta_{1d}\left(\eta_{dx},\eta_{dy}\right)\in R^{2}$, whereas as secondary objectives, $\eta_{2d}\left(\eta_{dz},\eta_{d\psi }\right)\in R^{2}$ was defined. It is important to mention that the secondary objectives will always be met whenever they do not conflict with the primary objectives. The desired task is defined as $\eta_{d}=[\begin{array}{cc} \eta_{1d} & \eta_{2d} \end{array}]\in R^{4}$ with respect to inertial reference frame <R>. The desired task and initial conditions for the controller are defined in Table 6 for the experiments.

**Figure 20:**
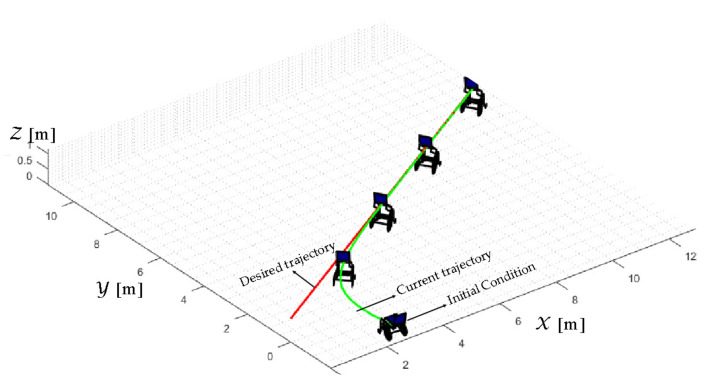
Stroboscopic movement of the human–robot system based on the real experimental data.

**Figure 21:**
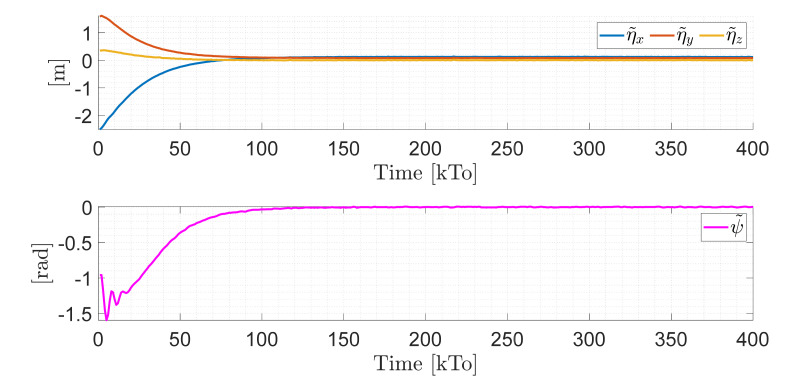
Time evolution of the control errors $\tilde{\eta }\left(kT_{0}\right)=\left({\tilde{\eta }}_{x},{\tilde{\eta }}_{y},{\tilde{\eta }}_{z},\tilde{\psi }\right)$.

**Figure 22:**
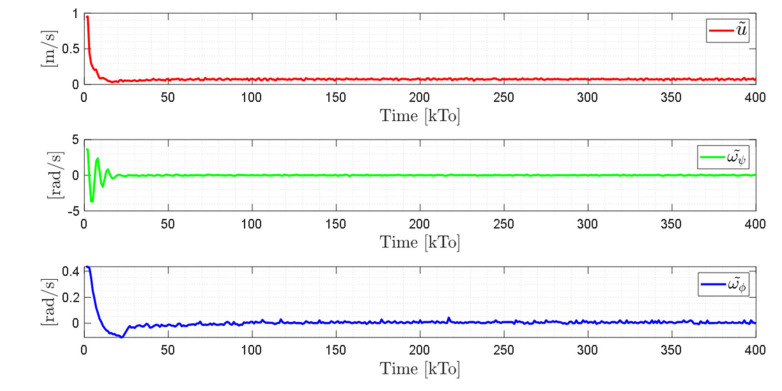
Time evolution of the control errors $\tilde{\mu }\left(kT_{0}\right)=\left(\tilde{u},{\tilde{\omega }}_{\psi },{\tilde{\omega }}_{\phi }\right)$.

**Figure 23:**
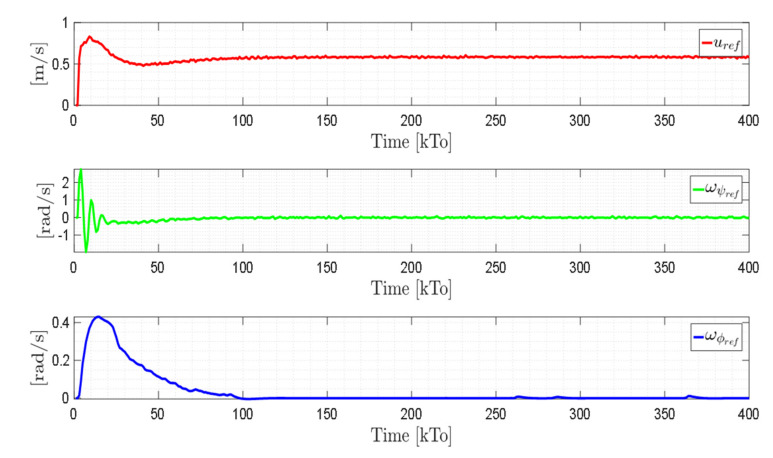
Velocity commands to the standing wheelchair $\mu_{ref}\left(kT_{0}\right)=\left(u_{ref},\omega_{\psi_{ref}},\omega_{\phi_{ref}}\right)$.

TRAJECTORY 1. The main results of the third experiment are observable in Figure 20, Figure 21, Figure 22 and Figure 23. Figure 20 presents the stroboscopic movement of the standing wheelchair system, based on real data from trajectory 1. It is shown that the controller above has an adequate performance. Figure 21 and Figure 22 show that the control errors $\tilde{\eta }\left({\tilde{\eta }}_{x},{\tilde{\eta }}_{y},{\tilde{\eta }}_{z},{\tilde{\eta }}_{\psi }\right)\in R^{4}$ and $\tilde{\mu }\left(\tilde{u},{\tilde{\omega }}_{\psi },{\tilde{\omega }}_{\phi }\right)\in R^{3}$, respectively, are ultimately bounded close to zero. We observe in Figure 21 that the errors tend to be zero when the robot is on the proposed trajectory. Figure 23 shows the control actions of the standing wheelchair robot.

**Figure 24:**
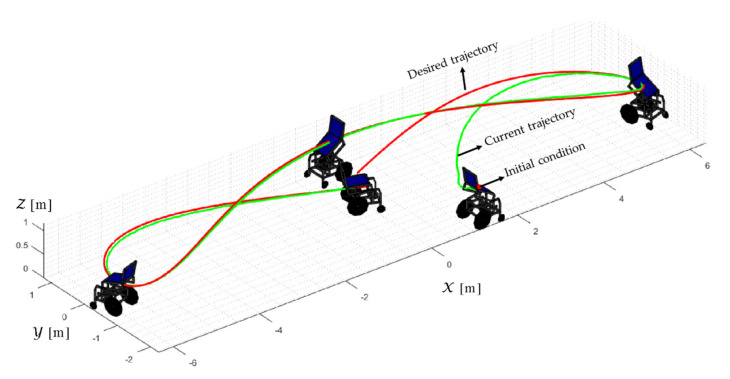
Stroboscopic movement of the human–robot system based on the real experimental data.

**Figure 25:**
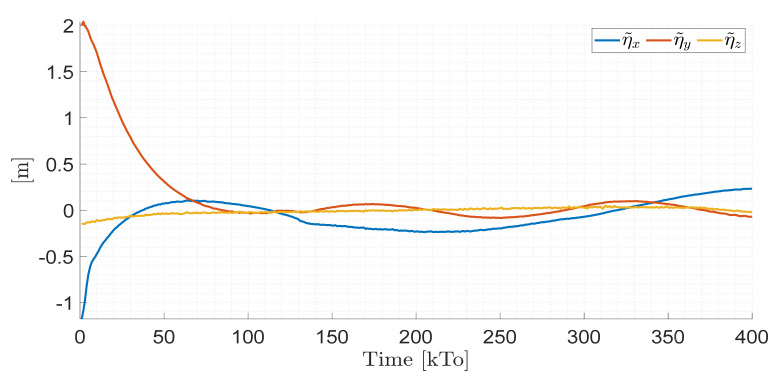
Time evolution of the control errors $\tilde{\eta }\left(kT_{0}\right)=\left({\tilde{\eta }}_{x},{\tilde{\eta }}_{y},{\tilde{\eta }}_{z}\right)$.

**Figure 26:**
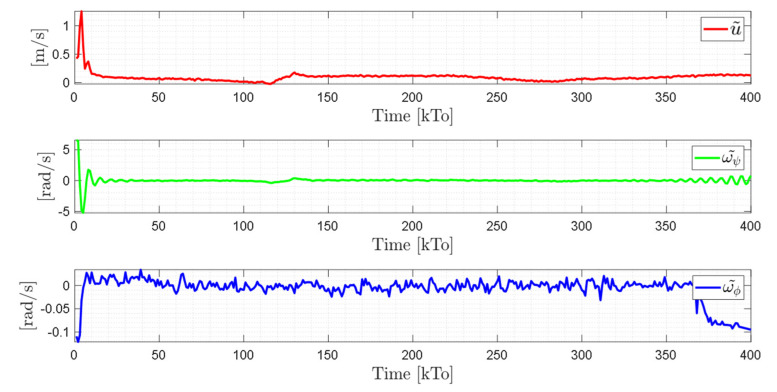
Time evolution of the control errors $\tilde{\mu }\left(kT_{0}\right)=\left(\tilde{u},{\tilde{\omega }}_{\psi },{\tilde{\omega }}_{\phi }\right)$.

**Figure 27:**
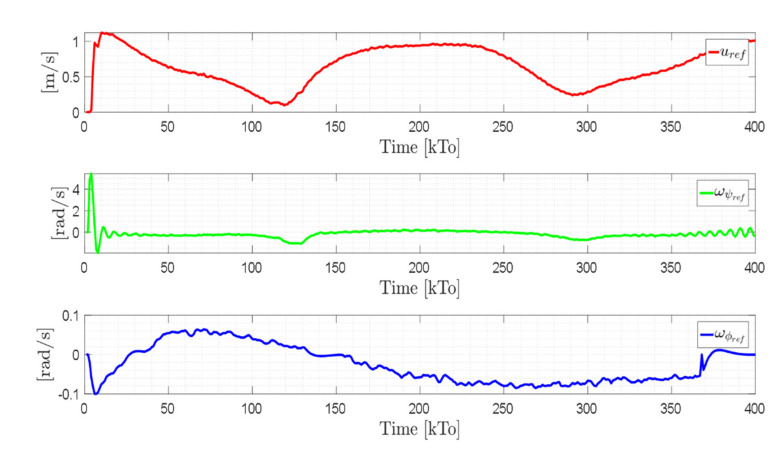
Velocity commands to the standing wheelchair $\mu_{ref}\left(kT_{0}\right)=\left(u_{ref},\omega_{\psi_{ref}},\omega_{\phi_{ref}}\right)$.

TRAJECTORY 2. The results of the experiment are illustrated in Figure 24, Figure 25, Figure 26 and Figure 27. Figure 24 presents the stroboscopic movement of the standing human–wheelchair system, based on real data from trajectory 2. Figure 25 and Figure 26 show that the control errors are $\tilde{\eta }\left({\tilde{\eta }}_{x},{\tilde{\eta }}_{y},{\tilde{\eta }}_{z},{\tilde{\eta }}_{\psi }\right)\in R^{4}$ and $\tilde{\mu }\left(\tilde{u},{\tilde{\omega }}_{\psi },{\tilde{\omega }}_{\phi }\right)\in R^{3}$, respectively, which are limited to values close to zero, i.e., achieving final characteristics errors $\max\left|\tilde{\eta }\left(kT_{0}\right)\right|<0.18\;[m]$. Figure 27 shows the control actions injected into the standing wheelchair robot during the experimental test. From the results obtained, the adequate performance of the proposed controller was verified.

TRAJECTORY 3. Finally, in order to evaluate the robustness of the proposed control scheme, an experimental test was performed with a 91 kg person on the bipedestation chair. The kinematic control law proposed in Equation (55) and the dynamic compensation proposed in Equation (64) were implemented. For the dynamic compensation, the dynamic parameters obtained for a 75 kg person were considered, as shown in Table 2. The experimental test dealt with the follow-up of a trajectory that best excited the dynamics of the robotic system. The desired trajectory selected is described by $\eta_{dx}=3sin(0.017\pi kT_{0})$, $\eta_{dy}=2sin(0.1kT_{0})$, $\eta_{dz}=0.6+0.2sin\left(0.1kT_{0}\right)$, and $\eta_{d\psi }=\tan^{-1}\left({\dot{\eta }}_{dy}/{\dot{\eta }}_{dx}\right)$, while, the initial conditions of the robotic system were defined as: $\eta_{0x}=1\;[m]$, $\eta_{0y}=-1\;[m]$, $\eta_{0z}=0.5\;[m]$, and $\eta_{0\psi }=\frac{\pi }{16}\;[\mathrm{rad}]$.

**Figure 28:**
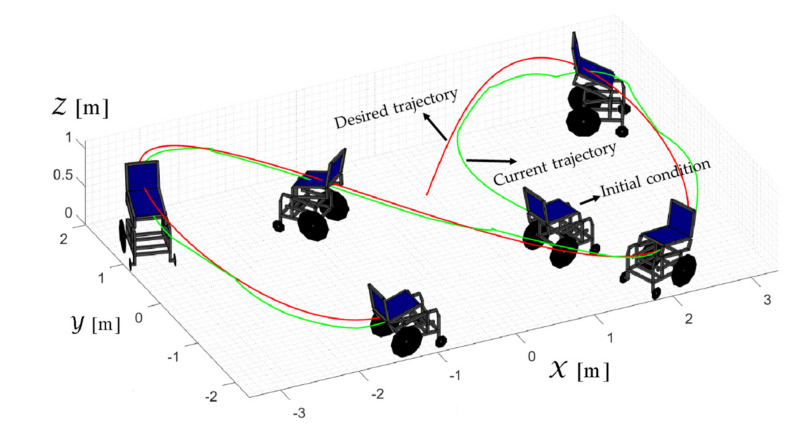
Stroboscopic movement of the human–robot system based on the real experimental data.

**Figure 29:**
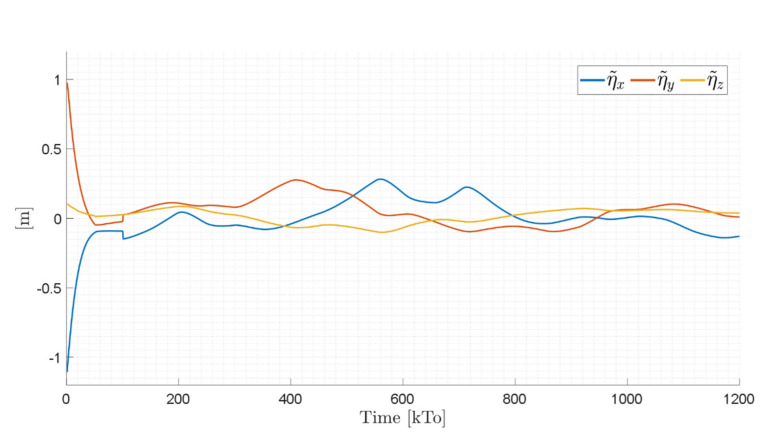
Time evolution of the control errors $\tilde{\eta }\left(kT_{0}\right)=\left({\tilde{\eta }}_{x},{\tilde{\eta }}_{y},{\tilde{\eta }}_{z}\right)$.

**Figure 30:**
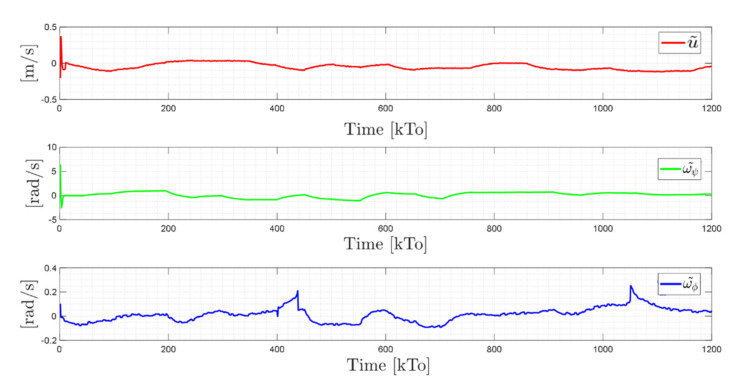
Time evolution of the control errors $\tilde{\mu }\left(kT_{0}\right)=\left(\tilde{u},{\tilde{\omega }}_{\psi },{\tilde{\omega }}_{\phi }\right)$.

**Figure 31:**
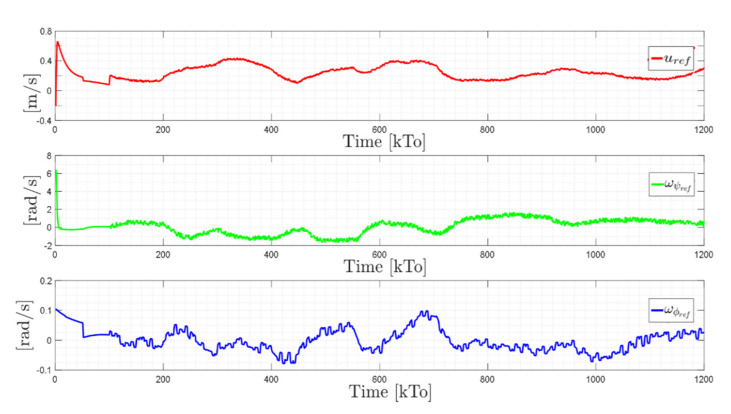
Velocity commands to the standing wheelchair $\mu_{ref}\left(kT_{0}\right)=\left(u_{ref},\omega_{\psi_{ref}},\omega_{\phi_{ref}}\right)$.

The results of the final experiment are shown in Figure 28, Figure 29, Figure 30 and Figure 31. The stroboscopic movement of the human–wheelchair system, based on real experimental data, is shown in Figure 28. Figure 29 and Figure 30 show that the control errors are $\tilde{\eta }\left({\tilde{\eta }}_{x},{\tilde{\eta }}_{y},{\tilde{\eta }}_{z},{\tilde{\eta }}_{\psi }\right)\in R^{4}$and $\tilde{\mu }\left(\tilde{u},{\tilde{\omega }}_{\psi },{\tilde{\omega }}_{\phi }\right)\in R^{3}$, respectively, which are limited to values close to zero, i.e., achieving final characteristics errors $\max\left|\tilde{\eta }\left(kT_{0}\right)\right|<0.29\;[m]$. Finally, Figure 31 shows the maneuverability velocities applied to the robotic systems.

The proper performance of the proposed controllers was verified through six experimental tests, using three different control laws (the controller design was presented in Section 3.2). The adequate performance of the control scheme proposed for the experimental robot showed that the standing wheelchair is capable of following the desired trajectory, compensating the dynamic effects. The latter can be presented by a change in the user’s position when using the standing wheelchair robot, or due to irregularities in the contact surface.

It should be emphasized that the first five experiments were carried out with a 75 kg person mounted on the standing chair, whereas the sixth experiment was carried out with a 91 kg person. From the results obtained experimentally, it can be concluded that in all experiments, the control error converges to values close to zero. Therefore, the control error $\left\|\tilde{\eta }\left(kT_{0}\right)\right\|$ will be bounded, provided that the control error $\left\|\tilde{\mu }\left(kT_{0}\right)\right\|$ is bounded. The control error $\tilde{\mu }\left(kT_{0}\right)$ is different from zero when experimental tests are performed with a person with a different weight than the weight used in the identification process of the dynamic parameters of the model. The same occurs when the surface on which the experimental tests are performed is different from the surface used in the identification of the dynamic parameters. However, the control error $\left\|\tilde{\eta }\left(kT_{0}\right)\right\|$ will be bounded as a function of the velocity error value $\left\|\tilde{\mu }\left(kT_{0}\right)\right\|\leq k_{\tilde{\mu }}$. This analysis is supported by the results obtained and the robustness analysis described in Section 3.6, specifically by Equation (70). Therefore, from the results obtained in this work, it is feasible to propose a control scheme with adaptive dynamic compensation for the human–wheelchair system.

## 5. Conclusions

In this work, a control scheme to solve the problem of autonomous movement of a standing wheelchair was presented. The design of control scheme consists of two cascaded subsystems: (1) Unified kinematic controller, in charge of fulfilling the task objective, i.e., the path following and positioning of standing wheelchair; and (2) Dynamic controller, in charge of compensating the dynamics of a standing wheelchair system. Sampling theory and Markov properties were considered for the design of the proposed controllers. Obtaining the kinematic and dynamic model of the standing wheelchair robot allowed us to obtain the design of the proposed controllers. In addition, the robustness and stability analysis of the proposed controller design demonstrated that the control error was stable. Finally, it was confirmed that the real experimental tests showed an adequate performance of the proposed controller. Future work deals with the development of an interactive and immersive 3D virtual environment to create a rehabilitation system for people with motor disabilities in lower extremities. The virtual system will receive real data from the robotic system, in order to present a real-time interaction between the standing wheelchair robot and the virtual environment.

## Figures and Tables

**Table 1:** Evolution of the control error ${\tilde{\eta }}_{x}$ in $kT_{0}$ instants of time.

*k*	${\tilde{\eta }}_{x}\left(k+1\right)$	$w_{x}{\tilde{\eta }}_{x}\left(k\right)$
1	${\tilde{\eta }}_{x}\left(2\right)$	$w_{x}{\tilde{\eta }}_{x}\left(1\right)$
2	${\tilde{\eta }}_{x}\left(3\right)$	$w_{x}{\tilde{\eta }}_{x}\left(2\right)=w_{x}^{2}{\tilde{\eta }}_{x}\left(1\right)$
3	${\tilde{\eta }}_{x}\left(4\right)$	$w_{x}{\tilde{\eta }}_{x}\left(3\right)=w_{x}^{3}{\tilde{\eta }}_{x}\left(1\right)$
⋮	⋮	⋮
*n*	${\tilde{\eta }}_{x}\left(n+1\right)$	$w_{x}{\tilde{\eta }}_{x}\left(n\right)=w_{x}^{n}{\tilde{\eta }}_{x}\left(1\right)$

**Table 2:** Dynamic parameters ς of the standing human–wheelchair system for a person of 75 kg.

System	Dynamic Parameters							
**Mobile Platform**	$\varsigma_{1}$	0.0987	$\varsigma_{2}$	0.0046	$\varsigma_{3}$	0.0986	$\varsigma_{4}$	−0.0014
	$\varsigma_{5}$	0.0987	$\varsigma_{6}$	−0.0001	$\varsigma_{7}$	0.0987	$\varsigma_{8}$	0.0032
	$\varsigma_{9}$	0.9214	$\varsigma_{10}$	0.0986	$\varsigma_{11}$	−0.0019	$\varsigma_{12}$	0.9582
**Bipedestation**	$\varsigma_{13}$	0.1885	$\varsigma_{14}$	0.0214	$\varsigma_{15}$	−0.0001	$\varsigma_{16}$	1.00
	$\varsigma_{17}$	0.0003	$\varsigma_{18}$	−0.0085	$\varsigma_{19}$	−0.0004	$\varsigma_{20}$	0.0229
	$\varsigma_{21}$	0.0005	$\varsigma_{22}$	−0.0038				

**Table 3:** Proposed gain values for the control scheme.

Gain Matrix	Gain	Value	Gain	Value	Gain	Value	Gain	Value
$W_{1}=\mathrm{diag}\left(\frac{w_{\eta_{1i}}}{k_{\eta_{1i}}+\left|\Delta \eta_{1i}\left(k\right)\right|}\right)$ $\in R^{2x2}$	$w_{\eta x}$	0.6	$k_{\eta x}$	1.5	$w_{\eta y}$	0.6	$k_{\eta y}$	1.5
$W_{2}=\mathrm{diag}\left(\frac{w_{\eta_{2i}}}{k_{\eta_{2i}}+\left|\Delta \eta_{2i}\left(k\right)\right|}\right)$ $\in R^{2x2}$	$w_{\eta z}$	0.7	$k_{\eta z}$	1.5	$w_{\eta \psi }$	0.5	$k_{\eta \psi }$	2.5
$W_{\mu }=\mathrm{diag}\left(\frac{w_{\tilde{\mu }i}}{k_{\tilde{\mu }i}+\left|\Delta {\tilde{\mu }}_{i}\left(k\right)\right|}\right)$ $\in R^{3x3}$	$w_{\tilde{u}}$	0.6	$k_{\tilde{u}}$	1.75	$w_{{\tilde{\omega }}_{\psi }}$	0.5	$k_{{\tilde{\omega }}_{\psi }}$	1.2
	$w_{{\tilde{\omega }}_{\varphi }}$	0.5	$k_{{\tilde{\omega }}_{\varphi }}$	1.2				

**Table 4:** Desired task and initial parameters.

Initial Conditions				Desired Task	
$\eta_{0x}$	−1.0 [m]	$u_{0}$	0 [m/s]	$\eta_{dx}$	0.1 [m]
$\eta_{0y}$	1.0 [m]	$\omega_{\psi 0}$	0 [rad/s]	$\eta_{dy}$	0.1 [m]
$\eta_{0z}$	0.47 [m]	$\omega_{\phi 0}$	0 [rad/s]	$\eta_{dz}$	‒
$\eta_{0\psi }$	−π/4 [rad]	‒	‒	$\eta_{d\psi }$	‒

**Table 5:** Desired task and initial parameters.

Initial Conditions				Desired Task	
$\eta_{0x}$	−1.0 [m]	$u_{0}$	0 [m/s]	$\eta_{dx}$	‒
$\eta_{0y}$	1.0 [m]	$\omega_{\psi 0}$	0 [rad/s]	$\eta_{dy}$	‒
$\eta_{0z}$	0.47 [m]	$\omega_{\phi 0}$	0 [rad/s]	$\eta_{dz}$	0.87 [m]
$\eta_{0\psi }$	−π/4 [rad]	‒	‒	$\eta_{d\psi }$	π/2 [rad]

**Table 6:** Desired trajectory and initial parameters.

Trajectory 1				Trajectory 2			
Initial Conditions		Desired Task		Initial Conditions		Desired Task	
$\eta_{0x}$	2.5 [m]	$\eta_{dx}$	$0.1kT_{0}$	$\eta_{0x}$	1 [m]	$\eta_{dx}$	$6sin(0.05\pi kT_{0})$
$\eta_{0y}$	−1.7 [m]	$\eta_{dy}$	$0.1kT_{0}$	$\eta_{0y}$	−2 [m]	$\eta_{dy}$	$sin(0.4kT_{0})$
$\eta_{0z}$	0.47 [m]	$\eta_{dz}$	$0.6+0.09sin\left(0.1kT_{0}\right)$	$\eta_{0z}$	0.47 [m]	$\eta_{dz}$	$0.6+0.2sin\left(0.1kT_{0}\right)$
$\eta_{0\psi }$	1.5 [rad]	$\eta_{d\psi }$	$\tan^{-1}\left({\dot{\eta }}_{dy}/{\dot{\eta }}_{dx}\right)$	$\eta_{0\psi }$	0 [rad]	$\eta_{d\psi }$	$\tan^{-1}\left({\dot{\eta }}_{dy}/{\dot{\eta }}_{dx}\right)$

## Data Availability

Not applicable.

## Appendix A

Dynamic Parameters of the Mobile Platform System $\varsigma_{1}=\frac{2K_{DT}K_{pa}+m_{w}R_{pa}r}{2K_{pa}K_{PT}}$; $\varsigma_{2}=\frac{R_{pa}r}{2K_{pa}K_{PT}}$; $\varsigma_{3}=-\frac{m_{w}R_{pa}br}{K_{pa}K_{PT}}$; $\varsigma_{4}=-\frac{R_{pa}br}{K_{pa}K_{PT}}$; $\varsigma_{5}=-\frac{m_{w}R_{pa}br}{2K_{pa}K_{PR}R}$ $\varsigma_{6}=-\frac{R_{pa}br}{2K_{pa}K_{PR}R}$ $\varsigma_{7}=\frac{2K_{DR}K_{pa}R+m_{w}R_{pa}r+m_{w}2R_{pa}a^{2}r+m_{w}2R_{pa}b^{2}r}{2K_{pa}K_{PT}R}$; $\varsigma_{8}=\frac{2R_{pa}r+2R_{pa}a^{2}r+2R_{pa}b^{2}r}{2K_{pa}K_{PT}R}$;

$\varsigma_{9}=\frac{K_{pb}+K_{PT}r}{K_{PT}r}$; $\varsigma_{10}=-\frac{m_{w}R_{pa}ar}{K_{pa}K_{PT}}$; $\varsigma_{11}=-\frac{R_{pa}ar}{K_{pa}K_{PT}}$; and $\varsigma_{12}=\frac{K_{PR}rK_{pb}R}{K_{PR}r}$

## Appendix B

Dynamic Parameters of the Standing System

$\varsigma_{13}=\frac{K_{d}}{K_{p}}$; $\varsigma_{14}=\frac{m_{h}K_{z}b^{2}R_{a}K_{l}}{K_{a}K_{h}K_{p}T_{a}}$; $\varsigma_{15}=\frac{m_{h}K_{z}^{2}b^{2}R_{a}}{K_{a}K_{h}K_{p}T_{a}}$; $\varsigma_{16}=\frac{K_{b}+K_{p}}{K_{p}}$; $\varsigma_{17}=\frac{m_{h}b^{2}R_{a}K_{h}}{K_{p}K_{a}K_{h}^{2}T_{a}\sin\left(T_{b}\right)\cos\left(T_{b}\right)}$

$\varsigma_{18}=-\frac{m_{h}b^{2}R_{a}K_{h}K_{z}K_{l}}{K_{p}K_{a}K_{h}^{2}T_{a}\sin\left(T_{b}\right)\cos\left(T_{b}\right)}$; $\varsigma_{19}=-\frac{m_{h}b^{2}R_{a}K_{h}K_{z}^{2}}{K_{p}K_{a}K_{h}^{2}T_{a}\sin\left(T_{b}\right)\cos\left(T_{b}\right)}$; $\varsigma_{20}=-\frac{m_{h}b^{2}R_{a}K_{h}K_{l}}{K_{p}^{2}K_{a}K_{h}T_{a}\sin\left(T_{b}\right)\cos\left(T_{b}\right)}$$\varsigma_{21}=-\frac{m_{h}b^{2}R_{a}K_{z}^{2}}{K_{p}^{2}K_{a}K_{h}T_{a}\sin\left(T_{b}\right)\cos\left(T_{b}\right)}$; and $\varsigma_{22}=\frac{m_{h}bR_{a}}{K_{a}K_{p}T_{a}\sin\left(T_{b}\right)\cos\left(T_{b}\right)}$.

Constant measured values and calculations through mathematical relationships: $T_{a}=412.021/8$; $T_{b}=0.155$; $T_{c}=420$; $T_{d}=0.2070$; $T_{e}=170$; $T_{f}=55$; $K_{z}=0.4$; $K_{l}=0.399$; $K_{h}=75.8436$; and $\phi_{e}=1.0509$.
